# Molecular Models of Atomically Dispersed Uranium at MoS_2_ Surfaces Reveal Cooperative Mechanism of Water Reduction

**DOI:** 10.1021/jacs.4c05002

**Published:** 2024-07-10

**Authors:** Kamaless Patra, William W. Brennessel, Ellen M. Matson

**Affiliations:** Department of Chemistry, University of Rochester, Rochester, New York 14627, United States

## Abstract

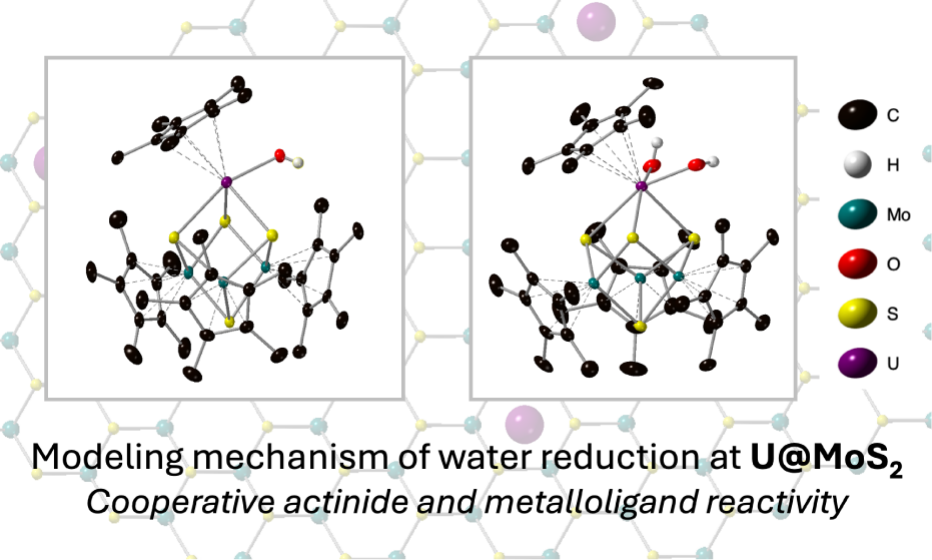

Single atoms of uranium supported on molybdenum sulfide surfaces (U@MoS_2_) have been recently demonstrated to facilitate the hydrogen evolution reaction (HER) through electrocatalysis. Theoretical calculations have predicted uranium hydroxide moieties bound to edge-sulfur atoms of MoS_2_ as a proposed transition state involved in the HER process. However, the isolation of relevant intermediates involved in this process remains a challenge, rendering mechanistic hypotheses unverified. The present work describes the isolation and characterization of a uranium-hydroxide intermediate on molybdenum sulfide surfaces using [(Cp*_3_Mo_3_S_4_)UCp*], a molecular model of a reduced uranium center supported at MoS_2_. Mechanistic investigations highlight the metalloligand cooperativity with uranium involved in the water activation pathway. The corresponding uranium-oxo analogue, [(Cp*_3_Mo_3_S_4_)Cp*U(=O)], was also accessed from the hydroxide cluster via hydrogen atom transfer and from [(Cp*_3_Mo_3_S_4_)UCp*] through an alternative direct oxygen atom transfer. These results provide an atomistic perspective on the reactivity of low-valent uranium at molybdenum sulfide surfaces toward water, modeling key intermediates associated with the HER of U@MoS_2_ catalysts.

## Introduction

The development of well-defined chemical uses for the long-lived radionuclides of the nuclear fuel cycle, namely the product of front-end enrichment processes (e.g., ^238^U), provides incentives for reprocessing these waste streams. Uranium is well-suited to participate in challenging multielectron molecular transformations, due to its ability to access a variety of oxidation states (U^2+^, U^3+^, U^4+^, U^5+^, U^6+^).^1−5^ The basis for the rich redox chemistry of this early actinide has been credited to the ability of 5f-orbitals to hybridize with 6d-orbitals.^6−9^ A number of examples of multielectron, catalytic small molecule activation processes by heterogeneous actinide-derived materials have been reported. This field of research traces back to a seminal breakthrough in 1909 when Haber first employed uranium as a heterogeneous catalyst for ammonia synthesis from N_2_ and H_2_ under high pressure and temperature.^10^ Subsequently, depleted uranium oxides have been used as catalysts for oxidative transformations, facilitating the oxidation of hydrocarbons as well as the partial oxidation of ethanol.^11−14^

Recent advancements have demonstrated the utilization of atomically disperse uranium centers anchored on redox-active surfaces as single-atom catalysts (SACs). These SACs have remarkable efficiency across a range of multielectron/multiproton-based chemical transformations. For instance, Zhai and co-workers reported uranium SACs supported on N-doped carbon, resulting in the formation of unprecedentedly high NH_3_ yields in electrochemical N_2_ reduction.^15^ Additionally, uranium atoms supported on main group and transition metal oxides have been extensively studied for catalytic oxidation reactions.^16^ Most similar to the work reported here, uranium SACs dispersed on MoS_2_ nanosheets (U@MoS_2_) have exhibited promising catalytic activity for electrocatalytic hydrogen evolution from water at low overpotential.^17^ However, to date, the intermediates involved in these catalytic pathways remain elusive, with mechanistic understanding derived primarily from predictions made by density functional theory (DFT) studies.

**Figure 1:**
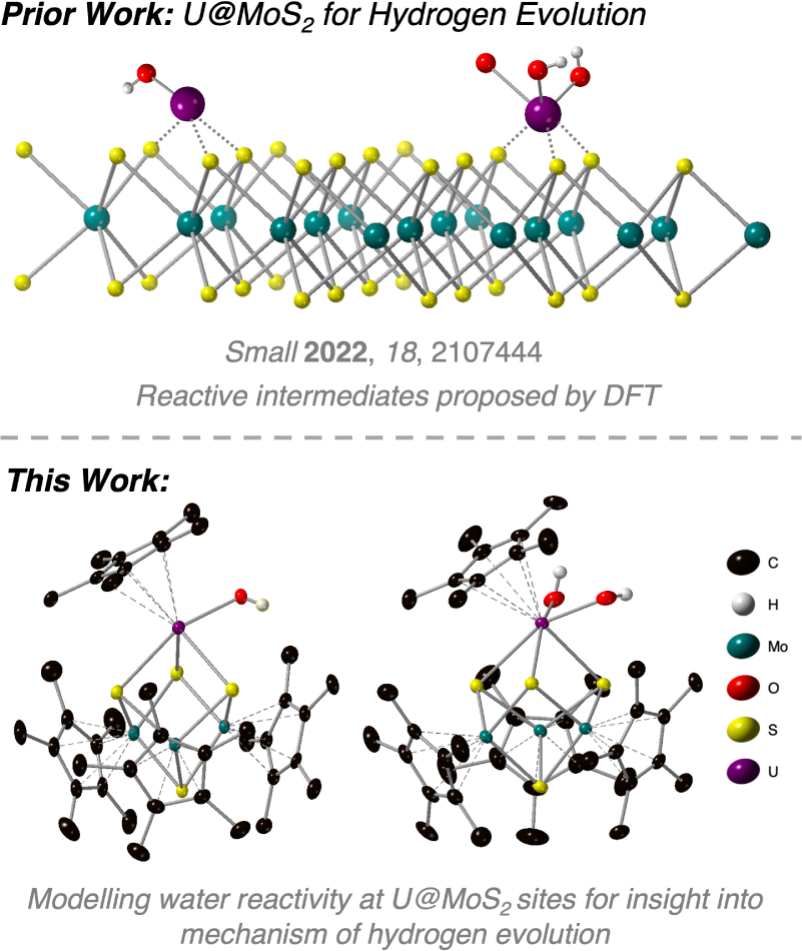
Reactivity of uranium on MoS_2_ surfaces toward water. The prior DFT model predicted a transition state for water activation on U@MoS_2_ (top). Molecular models describing relevant hydroxide intermediates from water reactivity (bottom).

Intrigued by the unique reactivity of uranium atoms deposited on redox active surfaces, our group has been investigating the coordination chemistry of a hemicubane thiomolybdate cluster, (Cp*_3_Mo_3_S_4_), with low-valent uranium (Figure 1).^18^ We view these heterometallic clusters as models for low-valent uranium ions deposited on the basal plane (i.e., 001 surface) of MoS_2_. Our team found that the coordination of the trisulfide face of the metalloligand (Cp*_3_Mo_3_S_4_) with Cp*UI_2_(THF)_3_ is associated with a charge transfer process, in which uranium is oxidized and the thiomolybdate assembly is reduced. Similar electron transfer reactions have been postulated as important in the installation of uranium ions at the surface of MoS_2_. Isolation of a redox series of the [(Cp*_3_Mo_3_S_4_)Cp*U]^*n*^ clusters (*n* = +2, +1, 0) revealed that reduction of the composite assembly serves to increase the affinity of the cluster for the actinide ion, providing insight into strategies for electrochemically cyclable actinide uptake at the basal plane of group(VI) chalcogenide surfaces.

Here, we utilize our mixed actinide-substituted thiomolybdate complexes to probe the mechanism of water activation at atomically dispersed uranium centers on molybdenum disulfide supports. The significant intermediates [(Cp*_3_Mo_3_S_4_)Cp*U(OH)_*n*_] (*n* = 1, 2) were isolated and characterized. Mechanistic investigations reveal that interactions between uranium and the sulfur atoms of the thiomolybdate cluster provide a cooperative pathway for water activation (i.e., O–H bond cleavage). Additionally, we explore the reactivity of the hydroxide cluster (Cp*_3_Mo_3_S_4_)Cp*U(OH) toward hydrogen atom transfer (HAT) reagents, resulting in the transient formation of the corresponding oxido-functionalized cluster, (Cp*_3_Mo_3_S_4_)Cp*U(=O). Access to the thermally unstable (Cp*_3_Mo_3_S_4_)Cp*U(=O) complex is confirmed through independent synthesis; O-atom transfer reactions involving styrene oxide reveal an alternative pathway to access the oxido-substituted species. Collectively, the results presented in this study provide a comprehensive understanding of the stochiometric reactivity of low-valent uranium on the molybdenum sulfide surface, modeling multiple significant intermediates involved in catalytic water reduction by uranium single-atom catalysts on MoS_2_ surfaces.

## Experimental Section

### Safety Considerations

Caution! Depleted uranium (primary isotope ^238^U) is a weak α-emitter (4.197 MeV) with a half-life of 4.47 × 10^9^ years; manipulations and reactions should be carried out in monitored fume hoods or in an inert atmosphere drybox in a radiation laboratory equipped with α- and β-counting equipment.

### General Considerations

All air- and moisture-sensitive manipulations were carried out using standard high vacuum line, Schlenk, or cannula techniques or in an MBraun inert atmosphere drybox containing an atmosphere of purified dinitrogen. Solvents for air- and moisture-sensitive manipulations were dried and deoxygenated using a glass contour solvent purification system (Pure Process Technology, LLC) and stored over activated 4 Å molecular sieves (Fisher Scientific) prior to use. Deuterated solvents for ^1^H NMR spectroscopy were purchased from Cambridge Isotope Laboratories and stored in the glovebox over activated 3 Å molecular sieves after three freeze–pump–thaw cycles. Deionized water was deoxygenated three times before being used to prepare a 0.1 M aqueous solution in anhydrous THF as a stock solution. This stock solution was then diluted to 0.05 M and deoxygenated again before being added to the cluster. Gomberg’s dimer was purchased from Sigma-Aldrich and recrystallized from anyhydrous diethyl ether prior to use. Anhydrous styrene oxide was purchased from Sigma-Aldrich, deoxygenated three times, and stored over 3 Å molecular sieves prior to its use. [(Cp*_3_Mo_3_S_4_)UCp*]^18^ and N_3_Mes^19^ (Mes = 2,4,6-trimethylphenyl) were synthesized following established procedures.

### Physical Measurements

^1^H NMR spectra were recorded at room temperature on a 400 MHz Bruker AVANCE spectrometer locked on the signal of deuterated solvents. All the chemical shifts are reported relative to the chosen deuterated solvent as a standard. Elemental analysis data were obtained from the Elemental Analysis Facility at the University of Rochester. Microanalysis samples were weighed with a PerkinElmer model AD6000 Autobalance, and their compositions were determined with a PerkinElmer 2400 Series II analyzer. Air-sensitive samples were handled in a VAC glovebox. Infrared (FT-IR, ATR) spectra of compounds were recorded on a Shimadzu IRAffinity-1 FT-IR spectrophotometer and are reported in wavenumbers (cm^–1^). The solution-phase FT-IR was performed under an inert atmosphere inside the glovebox using a 25 mm × 4 mm CaF_2_ supersealed liquid cell (International Crystal Laboratories). All solution-phase data were processed after subtracting the transmittance of the respective solvents.

### Single Crystal X-Ray Crystallography

The single crystals of (Cp*_3_**Mo**_**3**_**S**_**4**_)Cp*U(OH), (Cp*_3_**Mo**_**3**_**S**_**4**_)Cp*U(OH)_**2**_, and (Cp*_3_**Mo**_**3**_**S**_**4**_)Cp*U(=NMes) were placed on a nylon loop and mounted on a Rigaku XtaLAB Synergy-S Dualflex diffractometer equipped with a HyPix-6000HE HPC area detector for data collection at 100.00(10) K (Table S1). A preliminary set of cell constants and an orientation matrix were calculated from a small sampling of reflections. A short pre-experiment was run, from which an optimal data collection strategy was determined. The full data collection for all four complexes was carried out using a PhotonJet (Cu) X-ray source. After the intensity data were corrected for absorption, the final cell constants were calculated from the *xyz* centroids of the strong reflections from the actual data collections after integration. The structure was solved using SHELXT^20^ and refined using SHELXL.^21^ Most or all non-hydrogen atoms were assigned from the solution. Full-matrix least-squares/difference Fourier cycles were performed, which located any remaining non-hydrogen atoms. All the non-hydrogen atoms were refined with anisotropic displacement parameters. All the hydrogen atoms were placed in ideal positions and refined as riding atoms with relative isotropic displacement parameters.

### Synthesis of (Cp*_3_Mo_3_S_4_)Cp*U(OH)

A Schlenk tube was charged with a stir bar, (Cp*_3_Mo_3_S_4_)Cp*U (0.030 g, 0.025 mmol), and 5 mL of benzene. In a separate Schlenk tube, a deoxygenated solution of H_2_O in anhydrous THF (0.05 M, 0.6 mL) was prepared. The H_2_O solution was transferred slowly to the [Cp*_3_Mo_3_S_4_]Cp*U solution via Schlenk distillation and stirred. The solvent was removed immediately following the addition of water under reduced pressure and subsequently redissolved in toluene. The resulting toluene solution was filtered through a pipet containing filter paper to eliminate insoluble substances before being evaporated, yielding the title compound as a black powder. Yield = 0.028 g, 0.023 mmol, 93%. ^1^H NMR (400 MHz, C_6_D_6_) δ = 6.10 (45, 45H), −4.68 (47, 15H). IR (CaF_2_ liquid cell, C_6_D_6_): *v*(cm^–1^) = 3668 (m), 2978 (s), 2950 (s), 2901 (s), 2853 (s), 2716 (w), 1495 (m), 1470 (m), 1435 (m), 1375 (s), 1083 (w), 1017 (w). IR (ATR): *v*(cm^–1^) = 3672 (m), 2977 (m), 2954 (m), 2897 (s), 2846 (s), 2714 (w), 1494 (w), 1465 (m), 1428 (s), 1369 (s), 1077 (m), 1014 (s), 786 (s). Black crystals suitable for single crystal X-ray diffraction were grown from the concentrated toluene solution of the title compound at −30 °C. Anal. calcd for C_40_H_61_Mo_3_S_4_UO (mol. wt. 1212.046 g mol^–1^): C, 39.64%; H, 5.07%. Found: C, 40.05%; H, 5.13%.

### Synthesis of (Cp*_3_Mo_3_S_4_)Cp*U(OH)_2_

A J-young NMR tube was charged with [Cp*_3_Mo_3_S_4_]Cp*U(OH) (0.014 g, 0.012 mmol) and 1 mL of benzene. In a separate J-young NMR tube, a deoxygenated solution of H_2_O in anhydrous toluene (0.025 M, 0.6 mL) was prepared. The H_2_O solution was sonicated for 5 min and then slowly transferred to the [Cp*_3_Mo_3_S_4_]Cp*U(OH) solution via Schlenk distillation. The solvent was removed under vacuum. The ^1^H NMR was recorded in C_6_D_6_ solvent, which showed a mixture of products (see Supporting Information for details). Subsequently, the solvent was removed under reduced pressure, and the resulting residue was redissolved in toluene. The concentrated toluene solution of the title compound was stored at −30 °C for a week, yielding dark crystals suitable for single crystal X-ray diffraction. Upon crystallization, the compound’s solubility drastically decreased, posing challenges for further solution-phase characterization. IR (CaF_2_ liquid cell, C_6_D_6_): *v*(cm^–1^) = 3730 (w), 3704 (w), 3668 (m), 3645 (m), 3604 (w), 2978 (s), 2960 (s), 2907 (s), 2847 (s), 2722 (w), 1488 (s), 1470 (m), 1425 (s), 1375 (s), 1119 (w), 1071 (w), 1023 (w). Anal. calcd for C_40_H_62_Mo_3_S_4_UO_2_·(C_7_H_8_)_0.3_ (mol. wt. 1256.695 g mol^–1^): C, 40.24%; H, 5.17%. Found: C, 40.33%; H, 5.08%.

### Synthesis of (Cp*_3_Mo_3_S_4_)Cp*U(=O)

The title compound was synthesized by two procedures (methods A and B described below). Efforts to isolate this cluster were unsuccessful due to its instability. Elemental analysis and FT-IR data could not be recorded due to the extreme temperature sensitivity of the title compound.

### Method A. Hydrogen Atom Transfer from (Cp*_3_Mo_3_S_4_)Cp*U(OH) to Gomberg’s Dimer

A J-young NMR tube was charged with (Cp*_3_Mo_3_S_4_)Cp*U(OH) (0.014 g, 0.012 mmol) and 0.6 mL of C_6_D_6_. The solution was frozen after the dissolution of all solids. As the solution began to thaw, 0.2 mL of the C_6_D_6_ solution of Gomberg’s dimer (0.003 g, 0.006 mmol, 0.5 equiv) was added. The ^1^H NMR spectrum of the solution was taken immediately after the addition of Gomberg’s dimer. ^1^H NMR (400 MHz, C_6_D_6_) δ = 6.32 (41, 45H), 1.74 (28, 15H), 7.07 (Ph-*H*, Ph_3_CH).

### Method B. Oxygen Atom Transfer from Styrene Oxide to (Cp*_3_Mo_3_S_4_)UCp*

A J-young NMR tube was charged with (Cp*_3_Mo_3_S_4_)UCp* (0.015 g, 0.012 mmol) and 0.6 mL of toluene-d_8_. The solution was frozen after the dissolution of all solids. As the solution began to thaw, 0.2 mL of the toluene-d_8_ solution of styrene oxide (1.5 mg, 0.012 mmol, 1 equiv) was introduced to the solution via dropwise addition. The ^1^H NMR of the solution was monitored immediately after the addition. ^1^H NMR (400 MHz, toluene-d_8_) δ = 6.31 (41, 47H), 1.71 (24, 15H), 5.05 (C*H*_2_, styrene), 5.31 (C*H*_2_, styrene), 6.55 (C*H*, styrene), 7.19 (Ph-*H*, styrene).

### Synthesis of (Cp*_3_Mo_3_S_4_)Cp*U(=NMes)

A 20 mL scintillation vial was charged with (Cp*_3_Mo_3_S_4_)UCp* (0.020 g, 0.017 mmol) and 5 mL of toluene. In a separate vial, mesityl azide (N_3_Mes, 2.7 mg, 0.017 mmol, 1.0 equiv) was dissolved in 2 mL of toluene. The solution of N_3_Mes was added dropwise to the solution of (Cp*_3_Mo_3_S_4_)UCp* and swirled gently after the addition of each drop, and effervescence of *N*_2_(*g*) was immediately observed. The solution was then filtered through a pipet containing filter paper to remove insoluble byproducts. Removal of residual solvent under reduced pressure gave the title compound as a black powder. Yield = 0.021 g, 0.016 mmol, 94%. ^1^H NMR (400 MHz, C_6_D_6_) δ = 13.72 (45, 2H) 11.48 (40, 3H), 11.11 (40, 6H), 5.26 (46, 45H), 1.67 (46, 15H). Brown crystals suitable for single crystal X-ray diffraction were grown from the concentrated toluene solution of the title compound at −30 °C. Anal. calcd for C_49_H_71_Mo_3_S_4_NU·(KI)_0.4_ (mol. wt. 1394.634 g/mol): C, 42.20%; H, 5.13%; N, 1.00%. Found: C, 42.34%; H, 5.02%; N, 0.62%.

## Results and Discussion

### The Reactivity of (Cp*_3_Mo_3_S_4_)Cp*U toward Water

**Scheme 1:**
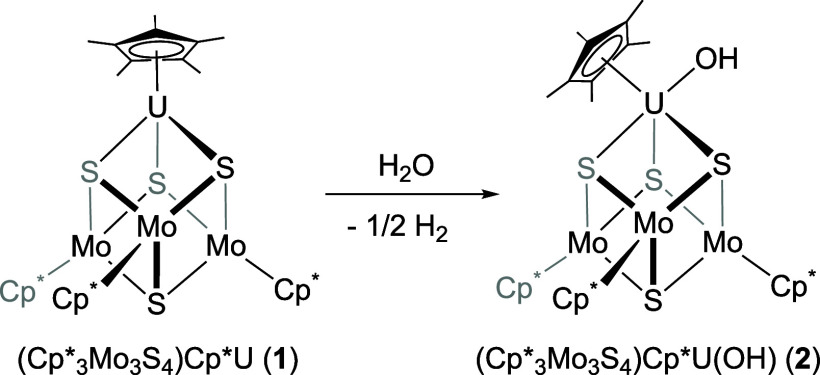
Synthesis of (Cp*_3_Mo_3_S_4_)Cp*U(OH) (**2**)

**Figure 2:**
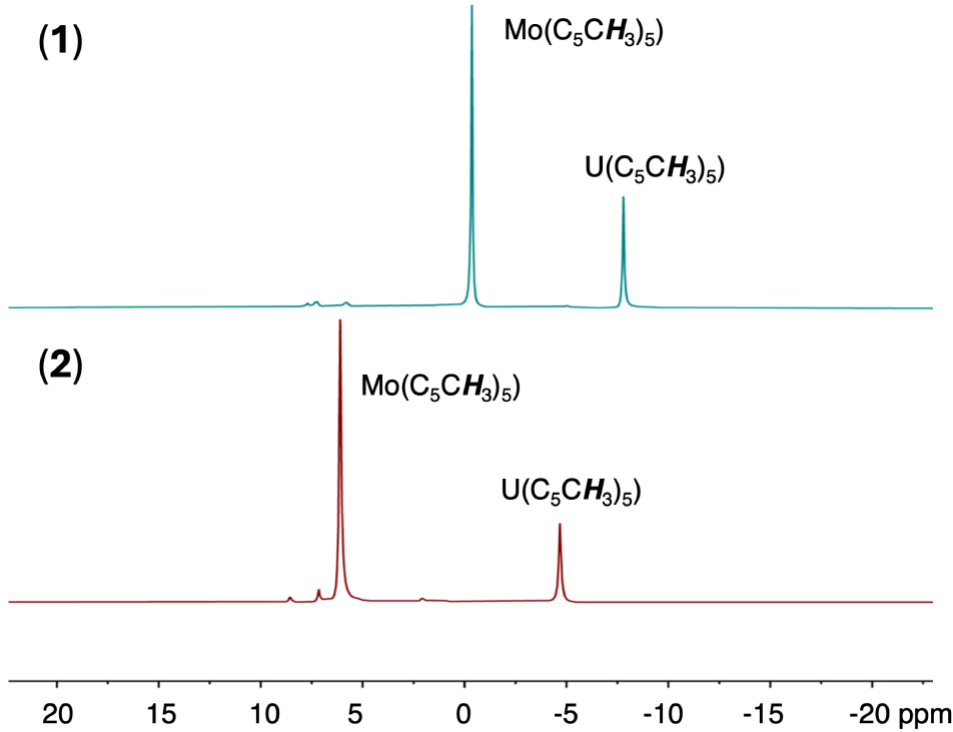
Stacked ^1^H NMR spectra (400 MHz) of complexes **1** (top, blue) and **2** (bottom, red) collected in C_6_D_6_ at room temperature (∼21 °C).

To interrogate the mechanism of water reduction at U@MoS_2_, we investigated the reactivity of the reduced uranium cluster (Cp*_3_Mo_3_S_4_)Cp*U (**1**) previously reported by our group.^18^ We present this compound as a molecular analogue of single U atom deposited on the basal plane of MoS_2_ nanosheets (U@MoS_2_). Upon addition of an equivalent of water (0.1 M solution in THF) to **1** in benzene, quantitative conversion of **1** to a single, paramagnetic product was observed (Scheme 1). Analysis of the crude reaction mixture by ^1^H NMR spectroscopy reveals a pattern of signals similar to that of **1**, with significant downfield shifts in the resonances corresponding to the protons of the Cp* ligand bound to uranium and the Cp* ligands of the thiomolybdate cluster (Figures 2 and S1). The chemical shift of the Mo-bound Cp* signal of the product resembles the value reported for the one-electron oxidized assembly, (Cp*_3_Mo_3_S_4_)Cp*UI,^18^ suggesting formation of an oxidized product. The proposed oxidation of the heterometallic assembly was accompanied by formation of ∼0.5 equiv of H_2_ (Figures S1 and S2; quantification of H_2_ in solution was determined by integration vs mesitylene as an internal standard). Based on this information, we proposed the formation of a uranium-hydroxyl cluster (Cp*_3_Mo_3_S_4_)Cp*U(OH) (**2**).

Analysis of the product by FTIR spectroscopy was performed on the C_6_D_6_ solution of the monohydroxide compound, **2** (Figure S3). The OH group of **2** was detected at 3668 cm^–1^, resembling the values reported by Meyer and co-workers for the uranium(IV) hydroxide complexes, ([(cyclen(Me)(^*t*-Bu,*t*-Bu^ArO)_3_U^IV^(OH)], (*v*(OH) = 3675 cm^–1^); [(^Ad,Me^ArO)_3_Mes)U(OH)], (*v*(OH) = 3659 and 3630 cm^–1^).^22,23^ The infrared spectrum of **2** was also recorded in the solid state using the attenuated total reflection (ATR) sampling technique; a similar absorption feature was observed (*v*(OH) = 3672 cm^–1^). The infrared spectra of the deuterated derivative of complex **2** were obtained following synthesis of **2-OD** via reaction of (Cp*_3_Mo_3_S_4_)Cp*U with D_2_O (*vide infra*). As expected, the *v*(OD) band appears at a lower energy (2705 cm^–1^; Figure S4), consistent with literature reports.^22^

**Figure 3:**
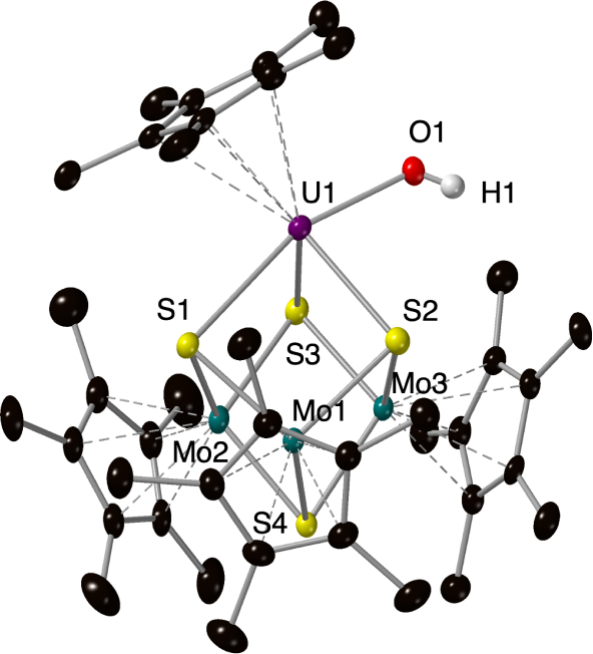
Molecular structure of **2** shown with 30% probability ellipsoids. Selected hydrogen atoms and solvent molecules have been removed for clarity. Key: H, white sphere; C, black; O, red; S, yellow; Mo, teal; U, purple.

Unambiguous confirmation of the formation of the purported uranium hydroxyl species was obtained through analysis of single crystals grown from the concentrated toluene solution of **2** at −30 °C (Figure 3, Table 1; see Table S1for structural parameters). The molecular structure of **2** reveals that the coordination environment of the uranium center is composed of the η^5^-Cp* ligand, the three sulfide ions which compose the face of the hemicubane metalloligand, and a new hydroxide ligand. The U–O bond distance in **2** is 2.123(5) Å; this distance is considerably elongated in comparison to previously reported low-valent uranium oxo complexes (*d* = 1.817(1)–1.917(6) Å^24−29^), supporting the assignment of this group as a hydroxyl substituent. The U–OH bond distance closely resembles that of the uranium(IV) hydroxide complex, ([(cyclen(Me)(*^t^*^–Bu,*t*-Bu^ArO)_3_U^IV^(OH)], (U–OH = 2.141(2) Å); [(^Ad,Me^ArO)_3_Mes)U–OH], U–OH = 2.106(2) Å), reported by Meyer and co-workers.^22,23^ Other uranium(IV) hydroxide complexes reported in the literature also exhibit U–O distances close to the value observed for **2** (e.g., ([U(OTf)_3_(OH)(py)_4_], U–OH = 2.040(2) Å;^30^ [Cp*_2_UCl(OH)(HNSPh_2_)], U–OH = 2.117(9) Å.^31^

**Table 1:** Pertinent Bond Distances and Angles of Complexes **2**, **3** and **5**

bond	(Cp*_3_Mo_3_S_4_)Cp*U(OH) (**2**)	(Cp*_3_Mo_3_S_4_)Cp*U(OH)_2_ (**3**)	(Cp*_3_Mo_3_S_4_)Cp*U(=NMes) (**5**)
U–Cp*_cent_	2.517(6) Å	2.519(2) Å	2.533(5) Å
U1–O1U1–O2	2.123(5) Å-	2.161(3) Å2.171(2) Å	-
U1=N1	-	-	2.019(7) Å
U1–N1–C41	-	-	176.9°
U–SX (*X* = 1, 2, 3)	2.6462(17)–2.7559(16) Å	2.7229(7)–2.8088(7) Å	2.645(2)–2.788(2) Å
MoX–SX (avg) (*X* = 1, 2, 3)	2.4030 Å	2.3701 Å	2.385 Å
MoX–S4 (avg) (*X* = 1, 2, 3)	2.3337 Å	2.3302 Å	2.325 Å
Mo–Mo (Å)	2.6881(6)–2.8843(7) Å	2.7655(3)–2.8952(3) Å	2.7221(11)–2.9364(10) Å
U–S_surf_	1.7087(12) Å	1.8622 (9) Å	1.7145 (15) Å

The extent of the interaction between the uranium center with the hemicubane thiomolybdate cluster is an important metric to examine in the structure of **2**. Prior work from our group has indicated that the distance between the trisulfide surface of the metalloligand and the uranium center is sensitive to the oxidation state of the cluster (e.g., (Cp*_3_**Mo**_**3**_**S**_**4**_)Cp*UI, U–S_surf_ = 1.6623(9) Å; (Cp*_3_**Mo**_**3**_**S**_**4**_)Cp*UI_**2**_, U–S_surf_ = 1.8554(6) Å; (Cp*_3_**Mo**_**3**_**S**_**4**_)Cp*U(NPh)_**2**_, U–S_surf_ = 2.0187(15) Å), suggesting extensive electronic communication between the low-valent uranium and its support.^18^ Similarly, several reports of uranium on MoS_2_ surfaces suggest that their formation is accompanied by a charge transfer between the uranium and the surface sulfur centers. This has been evidenced by X-ray absorption near edge structure spectroscopy and partial density of state (PDOS) analysis of corresponding theoretical models.^32−34^ To quantify the extent of interaction between the actinide and metalloligand, we inspected the distance between the uranium center and the centroid defining the plane of three μ^2^-bridged sulfur centers of Cp*_3_Mo_3_S_4_ (S_surf_). The U–S_surf_ distance in complex **2** is 1.7087(12) Å; this value resembles that observed in (Cp*_3_Mo_3_S_4_)Cp*UI (1.6623(9) Å), suggesting a similar charge state distribution over the metal ions in the two clusters.^18^

To confirm that water is the exclusive source of hydrogen atoms in the hydrogen evolution reaction (HER) of **1** with water, we conducted the same reaction with D_2_O under identical conditions as those of the H_2_O addition. Analysis of the reaction mixture using ^2^H NMR spectroscopy revealed the formation of D_2_, confirming that water indeed serves as the source of hydrogen following the reaction of water with **1**, rather than the solvents employed (Figure S5).

The mechanistic pathway for the reactivity of water with atomically dispersed, low-valent U@MoS_2_ nanosheets has been investigated through theoretical analysis and highlights the important role played by surface sulfur centers located at the surface of MoS_2_.^17^ These sites participate in cooperative O–H bond activation with uranium, thereby lowering the activation barrier for cleavage of the bond and serving as sites for H-atom capture. In this work, the formation of the U–OH complex from reduced species is rapid, with no intermediates observed, rendering the resolution of the kinetics of O–H bond activation and H-atom uptake at a surface sulfide challenging. To better understand the involvement of the redox-active thiomolybdate metalloligand (Cp*_3_Mo_3_S_4_) of our model cluster **1** in the water activation reactivity, we investigated the reactivity of [(^Mes^PDI^Me^)UCp*(THF)] with water. This compound features a different redox-active moiety in the form of a tridentate pyridine diimine (^Mes^PDI^Me^) ligand.^35^ Our interest in this complex stems from its broadly similar electronic structure to that of **1**; a low-valent uranium center is bound to an η^5^-Cp* moiety, and a reduced ^Mes^PDI^Me^ ligand. Indeed, analogous multielectron reactivity of the two complexes toward the activation of azobenzene has been reported; both [(^Mes^PDI^Me^)UCp*(THF)] and our model cluster **1** cleave the nitrogen–nitrogen double bond via a four-electron reduction of the substrate, resulting in the formation of a uranium bis-imido product.^35,36^ Notably in both examples, substrate activation results from electrons sourced from the reduced ligand and uranium center. Intriguingly, the addition of an equivalent of water to [(^Mes^PDI^Me^)UCp*(THF)] results in no reaction (Figure S6). The absence of signals attributed to uranium-bound THF molecules in ^1^H NMR spectrum of [(^Mes^PDI^Me^)UCp*(THF)] after the addition of water (THF-d_8_) in C_6_D_6_ is likely due to the rapid exchange of THF ligands with THF-d_8_ in solution. This observation underscores the specific role played by the thiomolybdate metalloligand in the activation of the O–H bond of water via **1**.

**Scheme 2:**
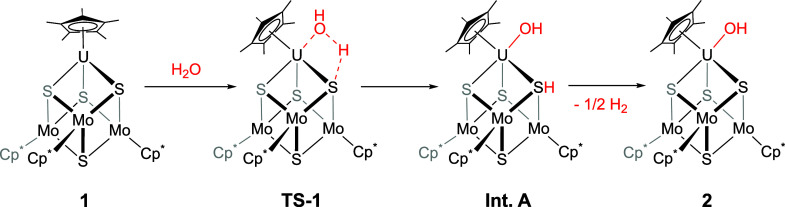
Proposed Mechanism for the Formation of **2**

With this information in hand, we propose a reaction pathway for the activation of water by **1** involving uranium–metalloligand cooperativity (Scheme 2). The proposed mechanism involves the homolytic cleavage of the O–H bond of water across the U–S interaction, proceeding through **TS-1**. In the next step of the proposed mechanism, we presume that the hydroxyl radical generated then adds to uranium, while the hydrogen atom is added to sulfur, forming intermediate **A**. In our deuterium-labeling study, the formation of D_2_ as the only byproduct from the reaction of **1** with D_2_O suggests that the D-atom coupling is very selective to couple with another D-atom in the solution, with no side reactivity observed with weak C–H bonds of the solvent. The S···H interaction in **A** undergoes facile homolytic bond cleavage, with the liberated H-atom coupling to an additional equivalent of H-atom in solution, resulting in the formation of half an equivalent of H_2_ and the final uranium-containing product (Cp*_3_Mo_3_S_4_)UCp*(OH) (**2**).

The steps involved in our proposed mechanistic pathway were further supported by the DFT calculations provided for the HER of U@MoS_2_ composite by Zhu and co-workers.^17^ The calculations were performed using a uranium single atom (UO_2_) decorated MoS_2_ slab. The analysis of DFT results predicts that the U=O on the MoS_2_ edge tends to polarize in the presence of water, leading to its dissociation and the formation of two hydroxyl ligands on uranium, proposed as a transition state for the HER pathway. This is followed by the transfer of an H atom from U–OH to an adjacent sulfur atom in MoS_2_. In the context of our study, we mirrored this pathway by presuming that in the absence of U=O, the H atom transfer could happen directly while activating the O–H bond of water to the adjacent surface sulfur atom of the metalloligand, and the hydroxyl ligand could be stabilized through the formation of a U–OH bond. The newly formed hydroxide ligand on uranium is reactive in the U@MoS_2_ composite, whereas it remains stable on the uranium–metalloligand complexes reported here. This stability/reactivity could be explained by considering the reactivity of surface/edge sulfur atoms on MoS_2_. A comparative theoretical analysis between the surface and edge sulfur centers in MoS_2_ nanosheets indicates that edge sulfur centers are more electron-rich and active as sites for uranium deposition under reductive conditions. This could also facilitate more efficient H atom capture from U–OH on edge sulfur centers compared to those on the surface of MoS_2_. Thus, we argue that the U–OH is likely to be stable on the surface while highly reactive on the edge. Another significant factor that influences the HER is the fraction coverage of the sulfide surface of the U@MoS_2_ composite by H atoms after their transfer. Greater surface coverage by H atoms makes hydrogen evolution less spontaneous and brings it closer to equilibrium (e.g., Δ*G*_H*_ = −0.40 eV and −0.14 eV for 25% and 50% H atom coverage on the U@MoS_2_ surface, respectively). In our study, with an approximate surface coverage of 33% by H atoms, Δ*G*_H*_ likely falls between the reported values for 25% and 50% coverage on the U@MoS_2_ composite surface. This relatively negative Δ*G*_H*_ may contribute to the rapid hydrogen evolution observed from the uranium-doped metalloligand.

The cooperative O–H bond activation by uranium and metalloligand constitutes a rare mode of small-molecule activation for actinide complexes. In the context of cooperative activation, the U–N cooperativity in uranium–amide and uranium–nitride species has previously been reported for small-molecule activations. For instance, uranium(IV) triamide complexes have been demonstrated to activate E–H bonds (where E = O, S) of alcohols and thiols across the uranium–amide bond, with the ligand amide functionality capturing and storing substrate proton.^37^ In another significant study, uranium nitride cooperativity was employed for the reversible activation of hydrogen.^38^ The uranium–metalloligand cooperative activation of a small-molecule substrate represents a unique mode of reactivity, where the H atom within the metalloligand is reactive and prone to further reactivity.

**Scheme 3:**
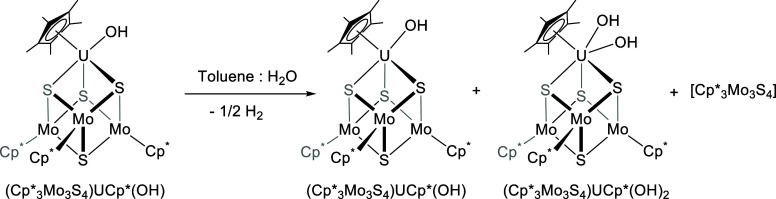
Reaction of (Cp*_3_Mo_3_S_4_)Cp*U(OH) (**2**) with Water

Considering the excess of remaining reducing equivalents in **2**, we hypothesized that the uranium hydroxide complex would be susceptible to reactivity with an additional equivalent of water. This could potentially result in the addition of another hydroxyl group to uranium, leading to the formation of a bis-hydroxide cluster similar to the bis-iodide cluster, [(Cp_3_Mo_3_S_4_)Cp*UI_2_], previously reported by our team.^18^ The addition of 1 equiv of water (in THF) to the C_6_D_6_ solution of **2** results in the formation of the uranium-free Cp_3_*Mo_3_S_4_ metalloligand. Surprisingly, when water was added to **2** in the presence of exclusively noncoordinating solvents, analysis of the crude sample by ^1^H NMR spectroscopy revealed the formation of a new product with paramagnetically shifted and broadened signals (note that additional resonances are observed in this spectrum, assigned for **2**, U-free Cp*_3_Mo_3_S_4_; Scheme 3, Figure S7). Attempts to isolate the newly formed desired compound through sequential solvent washing proved unsuccessful due to its instability in all nonpolar organic solvents, resulting in additional decomposition.

**Figure 4:**
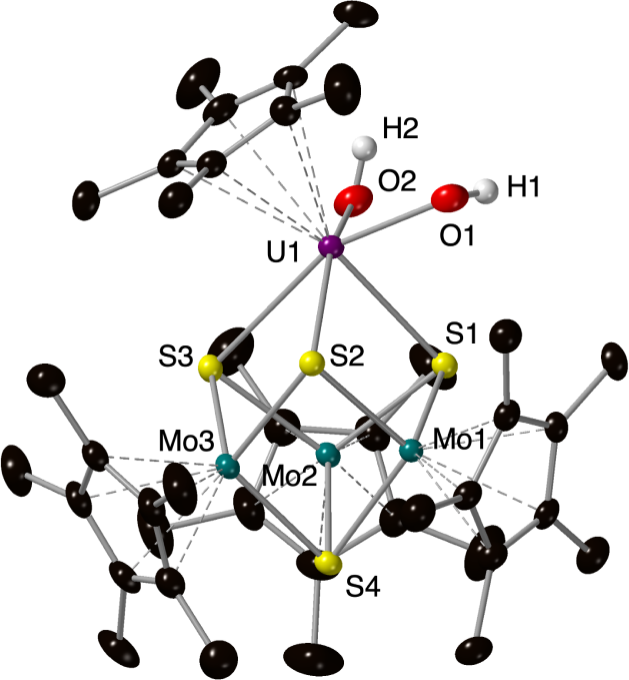
Molecular structure of **3** shown with 30% probability ellipsoids. Selected hydrogen atoms and solvent molecules have been removed for clarity. Key: H, white sphere; C, black; O, red; S, yellow; Mo, teal; U, purple.

Elucidation of the molecular structure of the product of the reaction of **2** with water was possible via selective crystallization from toluene at −30 °C. Dark crystals obtained from the crude reaction mixture were analyzed by single crystal X-ray diffraction, unambiguously confirming the generation of the bis-hydroxide cluster [(Cp_3_Mo_3_S_4_)Cp*U(OH)_2_] (**3**; Figure 4, Table 1; see Table S1for structural parameters). The molecular structure of **3** reveals that the coordination environment of the uranium center is similar to that observed in **2**, with the addition of a new second hydroxide ligand. The U–O bond distances in **3** are 2.171(3) and 2.161(3) Å, slightly elongated compared to those observed in **2** (2.123(5) Å). These U–O distances closely resemble those reported for the uranium-bis-hydroxide compound [(TPA)_2_U(OH)_2_]I_2_ (U–OH = 2.162(3) and 2.171(3) Å).^39^ A significant shortening of the Mo−μS_U_ (avg) distance is observed upon installation of a second hydroxide ligand at U (from 2.4030 to 2.3702 Å), consistent with the oxidation of the metalloligand. We note that this distance is slightly elongated compared to that in the analogous bis-iodide cluster [(Cp*_3_Mo_3_S_4_)Cp*UI_2_] (2.3644 Å), likely due to the steric bulk of the iodide.

The hydroxide ligands in **3** are positioned in a *cis* geometry (<O–U–O = 96.13(12)°). A notable difference between the reported example ([(TPA)_2_U(OH)_2_]I_2_) and **3** lies in the relative positions of the hydroxide substituents. In the case of [(TPA)_2_U(OH)_2_]I_2_, the bond angle between hydroxide moieties is much larger (<O–U–O = 134.69(5)°).^39^ The difference in observed geometries in these two complexes is likely a result of the three-dimensional structure of the molybdenum sulfide metalloligand in the case of **3**, which enforces a *cis* arrangement of the hydroxide moieties. The cis–bis-hydroxides are rare since they could easily be converted to corresponding oxos by releasing water or by transferring H atoms to the sulfur center of the metalloligand. Nonetheless, the finding of the bis-hydroxide intermediate with the model cluster aligns well with the theoretically predicted intermediates involved in the transition state of the HER reactivity of U@MoS_2_ with water (e.g., [MoS_2_]UO(OH)_2_).^17^

Existence of compound **3** in the solution phase was investigated through additional experiments. The ^1^H NMR analysis of the reaction mixture, with the sequential addition of a toluene-d_8_ and water binary mixture to the solution of the monohydroxide compound **2** in toluene-d_8_, revealed the formation of **3** and H_2_ (Figures S8 and S9). The chemical shift for the Mo–Cp* methyl protons observed in the reaction mixture matched exactly with those obtained from the crude solid product **3**. Additionally, the intensity of the signal assigned to product **3** increased along with the intensity of the hydrogen gas signal upon the addition of water (Figure S12), suggesting that the bis-hydroxide compound **3** is an immediate product following H_2_ liberation from the reaction of **2** and water.

The FT-IR spectrum of the reaction mixture of **2** and water in toluene-d_8_ shows a different absorption feature for the −OH group compared to the monohydroxide compound **2**, in the wavenumber range of 3500–4000 cm^–1^ (Figures S10 and S11). The analysis suggests the presence of a mixture of products, as also revealed by the ^1^H NMR spectrum of the crude reaction mixture. The presence of **2** in the mixture was confirmed by the *v*(OH) band at 3668 cm^–1^. In addition, new bands were observed in the range of OH absorptions, centered at wavenumbers of 3604, 3645, 3704, and 3730 cm^–1^. It is reasonable to attribute the lower energy bands centered at 3604 and 3645 cm^–1^ to the *v*(OH) stretching of **3**. This bathochromic shift compared to that of **2** can be explained by the fact that the addition of another hydroxide ligand is followed by the oxidation of the cluster, which makes the uranium center in the bis-hydroxide compound **3** more acidic, causing the OH band to shift to lower energy. A similar bathochromic shift of the *v*(OH) band with the addition of a hydroxido ligand has been evidenced in the theoretically predicted and experimentally observed FT-IR spectra of transition metal hydroxide compounds.^40^ Indeed, DFT calculations on the uranium-bishydroxide compound (UO_2_(OH)_2_) predict two expected bands for *v*(OH) at wavenumbers ranging from 3700 to 3800 cm^–1^.^41^ In the FT-IR spectrum of the reaction mixture containing **3**, two additional bands observed at higher energy are likely attributed to cluster-free uranium hydroxide (Scheme S1).

The addition of a catalytic amount of water to complex **1** or **2** in C_6_D_6_ resulted in the formation of the uranium-free Cp*_3_Mo_3_S_4_ metalloligand in the reaction mixture, as evidenced by the ^1^H NMR spectrum, irrespective of the choice of conditions (Figure S12). It is likely attributed to either the overoxidation of both the uranium center and the metalloligand, or the loss of Cp* upon protonation by water, leading to the dissociation of uranium from the metalloligand. Current efforts in our laboratory are underway to improve association constants of the thiomolybdate metalloligand to uranium in water and will be the subject of future work.

### Formation of (Cp*_3_Mo_3_S_4_)Cp*U(=O)

**Scheme 4:**
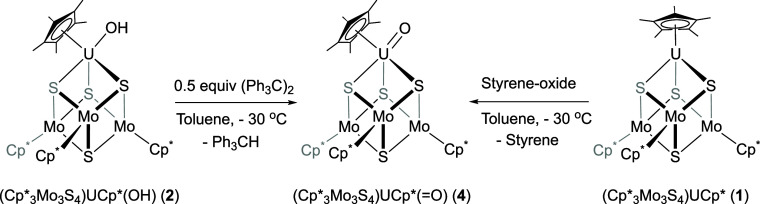
Synthesis of (Cp*_3_Mo_3_S_4_)Cp*U(=O) (**4**)

Uranium-oxo moieties have been cited as playing a pivotal role in the electrocatalytic hydrogen evolution reaction with water as the substrate.^17,22^ As such, after isolating the mono- and bis-hydroxide substituted uranium clusters, complex **2** and **3**, respectively, our focus shifted to understanding the ability of uranium single atom on MoS_2_ to form oxo ligands. We hypothesized that homolytic cleavage of the O–H bond of the hydroxyl ligand would result in the formation of the corresponding uranium-oxo cluster. In this context, we examined the reactivity of **2** toward the hydrogen atom transfer reagent, Gomberg’s dimer ((Ph_3_C)_2_; Scheme 4).^42^ The addition of a C_6_D_6_ solution containing 0.5 equiv of Gomberg’s to the C_6_D_6_ solution of **2** leads to an immediate darkening of the color of the solution. Analysis of the crude reaction mixture by ^1^H NMR spectroscopy revealed consumption of Gomberg’s dimer and the formation of Ph_3_CH, consistent with the successful transfer of an H atom from the U–OH species. Paramagnetically broadened resonances of the uranium-bound thiomolybdate cluster were observed at 1.73 ppm (U–Cp*, 15H) and 6.32 ppm (Mo–Cp*, 45H), which are shifted downfield from those of the starting material (Figure S13). Based on these results, we hypothesized that the desired uranium oxo complex, [(Cp*_3_Mo_3_S_4_)UCp*(=O)] (**4**), was formed under these conditions.

Hydrogen atom transfer (HAT) reactions have been extensively studied using transition metal aquo/hydroxides and amides, offering an alternative pathway for the synthesis of the corresponding oxo and imido congeners.^42−49^ Despite significant progress with transition metal complexes, HAT reactions involving actinides remain limited. The lack of understanding of HAT chemistry from uranium complexes is particularly significant, given uranium’s propensity for involvement in single-electron processes. In this context, Bart and co-workers have previously explored HAT reactions to convert uranium amides to uranium imido complexes.^50^ Similarly, Meyer and co-workers have reported the formation of uranium-oxo compounds from corresponding uranium-hydroxide compounds upon exposure to air;^23^ oxo formation likely proceeds through an HAT to O_2_ from the U–OH complex.

Complex **4** is quite unstable and readily decomposes in the solution and solid state, even at low temperatures. This fact made further isolation and structural characterization of **4** challenging. Confirmation of the formation of the uranium oxo cluster **4** was instead pursued via independent synthesis (e.g., O-atom transfer). Preliminary attempts at the O-atom transfer reaction of **1** utilizing well-known oxygen transfer reagents such as N_2_O, pyridine N-oxide, and iodosobenzene resulted in a significant amount of cluster decomposition in the form of the uranium-free metalloligand, Cp_3_*Mo_3_S_4_. However, oxidation of the low-valent uranium center via O-atom transfer from styrene oxide (Scheme 4) affords the generation of styrene (as characterized by ^1^H NMR spectroscopy), consistent with successful O-atom transfer. Analysis of the uranium-containing product via ^1^H NMR spectroscopy reveals paramagnetically broadened resonances at 6.31 and 1.71 ppm; the pattern of resonances formed is consistent with the formation of **4** (Figures S14 and S15).

To address the challenges posed by the instability of compound **4** toward structural characterization, we instead pursued the characterization of an isoelectronic model complex. Uranium imido compounds, featuring U=N bonds, have emerged as stable analogues of U=O in uranyl due to the isoelectronic nature of the imido ligand (NR_2_^2–^) with the oxo (O^2–^) ligand.^51,52^ The substituents of imido ligands enable exploration of steric and electronic effects on uranium–heteroatom multiple bonding, aspects not readily available in oxo compounds. Based on these considerations, we hypothesized that formation of a uranium imido analogue of **4** would offer enhanced stability, rendering the sample suitable for further structural analysis.

**Scheme 5:**
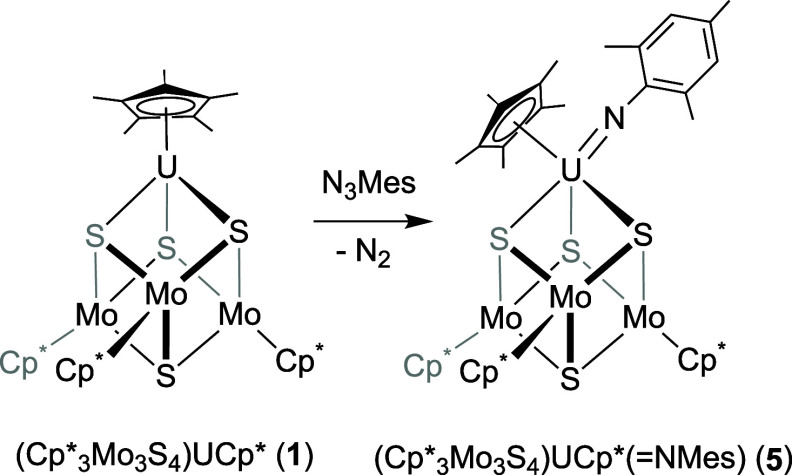
Synthesis of (Cp*_3_Mo_3_S_4_)Cp*U(=NMes) (**5**)

Synthesis of an imido-substituted cluster was attempted by treating the fully reduced cluster **1** with mesityl azide (Scheme 5). Upon addition of the azido substrate, immediate effervescence was observed consistent with the release of *N*_2_(*g*). The product, (Cp*_3_Mo_3_S_4_)Cp*U(=NMes) (**5**), is isolated in nearly quantitative yield (94%, see Experimental Section for more details). Initial characterization of **5** was performed using ^1^H NMR spectroscopy (Figure S17). The spectrum of **5** exhibited resonances assigned to Cp*-methyl protons on molybdenum at 5.26 ppm, which is shifted slightly upfield compared to that of **4**. However, the chemical shift of the Cp*-methyl protons in U–Cp* is observed at 1.67 ppm, similar to that observed for **4**, suggesting similar electronic environments at uranium for the two complexes (Figure S18). Signals corresponding to protons from the mesityl group exhibit an intensity ratio of 6:3:2. Specifically, the *ortho*-methyl protons of the mesityl group resonate at 11.11 ppm, while the *para*-methyl protons resonate at 11.48 ppm. Furthermore, the ring protons are assigned a resonance at 13.72 ppm. Similar paramagnetically shifted resonances have been observed for the protons of mesityl imido ligands in both the Tp*_2_U(=NMes)^53^ and ^Mes^PDI^Me^UI_2_(=NMes)(THF)^54^ complexes.

**Figure 5:**
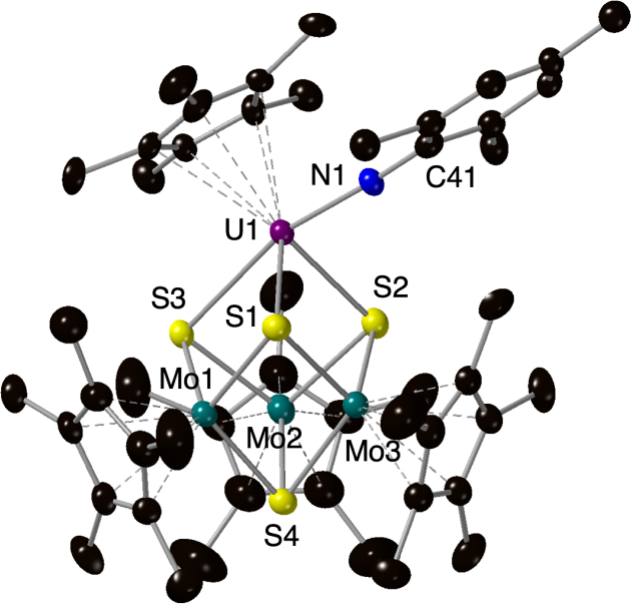
Molecular structure of (Cp*_3_Mo_3_S_4_)Cp*U(=NMes) (**5**) shown with 30% probability ellipsoids. Hydrogen atoms and solvent molecules contained within the unit cell have been removed for clarity. Key: H, white sphere; C, black; N, blue; S, yellow; Mo, teal; U, purple.

Complex **5** was structurally characterized by SCXRD (Figure 5, Table 1; see Table S1 for structural parameters). Dark brown, needle-shaped crystals of **5** were grown from a concentrated solution of the compound in toluene at −30 °C. The coordination environment of the uranium center contains a single mesityl imido substituent, the η^5^-Cp* ligand, and the three sulfide atoms, composing the face of the hemicubane thiomolybdate scaffold. The short U–N bond of the mesityl imido substituent is consistent with double bond character (2.019(7) Å) and is in good agreement with previously reported U(IV) imido complexes: ([η^5^-1,2,4-(Me_3_Si)_3_C_5_H_2_]_2_U=N(*p*-tolyl)(dmap), (2.021(5) Å);^55^ [η^5^-1,2,4-(Me_3_C)_3_C_5_H_2_]_2_U=N(*p*-tolyl)(dmap), (2.034(10) Å);^56^ [η^5^-1,3-(Me_3_C)_2_C_5_H_3_]_2_U=N(*p*-tolyl)(dmap) (2.002(3) Å);^57^ [η^5^-1,2,4-(Me_3_C)_3_C_5_H_2_]_2_U=N(*p*-tolyl) (1.988(5) Å).^27^

The U–S bond distances in **5** range from 2.645(2)–2.788(2) Å, with a U–Cp*_cent_ distance of 2.533(5) Å, which closely resemble those observed in **2**. The Mo−μS_U_ (avg) distance in **5** is 2.385 Å, significantly shorter than that observed in **2** (2.4030 Å). Considering the reflection of these bond parameters on uranium-oxo cluster **4**, we argue that similar U–S and U–Cp*_cent_ distances suggest that the metalloligand contributes more to the shared electron density than uranium during the conversion of (Cp*_3_Mo_3_S_4_)Cp*U(OH) to (Cp*_3_Mo_3_S_4_)Cp*U(=O). We inspected the distance between the uranium center and the centroid defining the plane of three μ^2^-bridged sulfide moieties of Cp*_3_Mo_3_S_4_ (S_surf_) (1.7145 (15) Å) to quantify the extent of interaction between the actinide and metalloligand surface. This distance is statistically similar to that of **2** (1.7087(12) Å), which is in contrast to the prior results that indicate the oxidation results in elongation of interactions.^18^ This anomaly could be explained by a minimal change in sterics brought by the additional ligand on uranium (U–S_surf_ = 1.8554(6) Å in (Cp*_3_Mo_3_S_4_)Cp*UI_2_ vs U–S_surf_ = 1.6623(9) Å in (Cp*_3_Mo_3_S_4_)Cp*UI). However, the similarity between the U–O distance in compound **2** and the U=NMes distance in compound **5** suggests a relatively weak U=O bond or the presence of a partially oxidized uranium center in **4**, which is consistent with its observed instability.

The findings with **5**, as the representative of the oxo-cluster **4**, have elucidated factors contributing to the instability of uranium oxo on the surface of the molybdenum sulfide metalloligand. This suggests that the presence of uranium oxo as a potential intermediate in our water activation pathway may be unlikely, in contrast to observations made during catalytic water activation with the molecular uranium complex [((^Ad,Me^ArO)_3_mes)U].^22^

## Conclusion

In summary, the work presented in this report explores the reactivity of low-valent uranium supported by the surface of a redox-active molybdenum sulfide metalloligand (**1**) toward water. Uranium and metalloligand constitute a cooperative pathway for water activation, as revealed by the mechanistic investigations. Notably, our findings underscore the stability of uranium hydroxides (**2** and **3**) on the trisulfide surface of the metalloligand, facilitating the isolation of these crucial intermediates, in contrast to their pronounced reactivity on the edge-site of the MoS_2_ composite. Moreover, the corresponding uranium-oxo analogue (**4**) was accessed via hydrogen atom transfer (HAT) and oxygen atom transfer (OAT) reactions and modeled using an isoelectronic monoimido cluster (**5**). Structural analysis of **5** elucidates factors contributing to the instability of uranium oxo on the metalloligand surface. We predict that the limitations of our model in stabilizing uranium oxo or uranyl derivatives possibly stem from weaker U–S_surf_ bonds compared to other reported analogous compounds featuring stronger U–X (X = N, O) bonds. Consequently, our ongoing investigations focus on modifying the metalloligand to enhance the stability of uranium-oxo compounds, paving the way for their application in uranyl chemistry and catalytic studies.

## Supporting Information Available

^1^H NMR and FT-IR spectra for all compounds (**2–5**) and selected crystallographic parameter for complexes **2, 3**, and **5**(PDF)
