# Supplementary material for: Molecular Models of Atomically Dispersed Uranium at MoS2 Surfaces Reveal Cooperative Mechanism of Water Reduction
Source: J Am Chem Soc. 2024 Jul 10;146(29):20147–57. doi: 10.1021/jacs.4c05002 (PMC11273346; doi:10.1021/jacs.4c05002)
Supplement: Supplementary file 1 — ja4c05002_si_001.pdf [file ja4c05002_si_001.pdf]

Electronic Supporting Information

Molecular Models of Atomically Dispersed Uranium at MoS_2 Surfaces Reveal Cooperative Mechanism of Water Reduction

Kamaless Patra, William W. Brennessel, and Ellen M. Matson*

Department of Chemistry, University of Rochester, Rochester, New York 14627, United States

* Corresponding author

SUPPORTING INFORMATION TABLE OF CONTENTS

Table S1. Crystallographic parameters for complexes $(Cp*_3Mo_3S_4)Cp*U(OH)$, $(Cp*_3Mo_3S_4)Cp*U(OH)_2$ and $(Cp*_3Mo_3S_4)Cp*U(=NMes)$ S3

Figure S1. ^{1}H NMR spectrum of $(Cp*_3Mo_3S_4)Cp*U(OH)$ in benzene-d_6 .... S4

Figure S2. ^{1}H NMR characterization of the evolved H_2 gas from the reaction mixture of $(Cp*_3Mo_3S_4)Cp*U$ and water in benzene-d_6 .... S4

Figure S3. Infrared spectrum of $(Cp*_3Mo_3S_4)Cp*U(OH)$ in benzene-d_6 .... S5

Figure S4. Stacked infrared spectra of $(Cp*_3Mo_3S_4)Cp*U(OH)$ and $(Cp*_3Mo_3S_4)Cp*U(OD)$ in benzene-d_6 .... S5

Figure S5. ^{2}H NMR characterization of the evolved D_2 gas from the reaction mixture of $(Cp*_3Mo_3S_4)Cp*U$ and D_2O in benzene-d_6 .... S6

Figure S6. Stacked ^{1}H NMR spectra of [(PDI)UCp*(THF)] and the reaction mixture of (PDI)UCp*(THF) and water (in THF-d_8) in benzene-d_6 .... S6

Figure S7. ^{1}H NMR spectra of the crude product obtained from the reaction of $(Cp*_3Mo_3S_4)UCp*(OH)$ with toluene-water binary mixture in benzene-d_6 .... S7

Figure S8-S9. Stacked ^{1}H NMR spectra of the reaction mixture containing $(Cp*_3Mo_3S_4)Cp*U(OH)$ and water in toluene-d_8 .... S8

Figure S10-S11. Stacked infrared spectra of $(Cp*_3Mo_3S_4)Cp*U(OH)$ and the crude reaction mixture containing $(Cp*_3Mo_3S_4)Cp*U(OH)$ and ~1.5 equiv of water in toluene-d_8 .... S9

Figure S12. ^{1}H NMR spectrum of the reaction mixture containing benzene-d_6 solutions of $(Cp*_3Mo_3S_4)Cp*U(OH)$ and $(Cp*_3Mo_3S_4)Cp*U(OH)$ with excess water S10

Figure S13-15. Individual and stacked ^{1}H NMR spectra of the reaction mixtures containing $(Cp*_3Mo_3S_4)Cp*U(OH)$ and Gomberg's dimer (**S13**), and $(Cp*_3Mo_3S_4)Cp*U$ and styrene-oxide in toluene-d_8 (**S14**) S10-11

Figure S16. Infrared spectrum of $(Cp*_3Mo_3S_4)Cp*U(=O)$ S12

Figure S17. ^{1}H NMR spectrum of $(Cp*_3Mo_3S_4)Cp*U(=NMes)$ in benzene-d_6 .... S13

Figure S18. Stacked ^{1}H NMR spectra of the reaction mixture containing C_6D_6 solution of $(Cp*_3Mo_3S_4)Cp*U(OH)$ and Gomberg's dimer and $(Cp*_3Mo_3S_4)Cp*U$ (=NMes) in benzene-d_6.. .... S13

Figure S19. Infrared spectrum of $(Cp*_3Mo_3S_4)Cp*U$. S14

Scheme 1. A possible decomposition pathway for the bis-hydroxide cluster $[(Cp*_3Mo_3S_4)Cp*U(OH)_2]$ S14

Table S1: Crystallographic parameters for molecular structures of complexes for **$(Cp^*_3Mo_3S_4)Cp^*U(OH)$**, **$(Cp^*_3Mo_3S_4)Cp^*U(=NMes)$** and **$(Cp^*_3Mo_3S_4)Cp^*U(OH)_2$**.

Compound	**$(Cp^*_3Mo_3S_4)Cp^*U(OH)$**	**$(Cp^*_3Mo_3S_4)Cp^*U(=NMes)$**	**$(Cp^*_3Mo_3S_4)Cp^*U(OH)_2$**
Empirical formula	$C_{47}H_{69}Mo_3OS_4U$	$C_{59.50}H_{83}Mo_3NS_4U$	$C_{53.21}H_{71.65}D_{4.76}Mo_3O_2S_4U$
Formula weight	1304.11	1466.35	1406.90
Temperature	100.00(10) K	100.00(10) K	100.00(10) K
Wavelength	1.54184 Å	1.54184 Å	1.54184 Å
Crystal system	triclinic	monoclinic	triclinic
Space group	*P*-1	*I*2/*a*	*P-1*
Unit cell dimensions	a = 11.2529(2) Å<br>b = 11.6796(3) Å<br>c = 19.1753(4) Å<br>α = 106.9151(18)°<br>β = 93.1763(15)°<br>γ = 96.8077(16)°	a = 24.0745(3) Å<br>b = 12.5265(2) Å<br>c = 37.8374(4) Å<br>α = 90°<br>β = 92.1880(10)°<br>γ = 90°	a = 11.63214(11) Å<br>b = 12.19606(11) Å<br>c = 19.30995(14) Å<br>α = 102.1358(7)°<br>β = 94.4320(7)°<br>γ = 100.3656(8)°
Volume / $Å^3$	2383.42(8)	11402.3(3) $Å^3$	2615.56(4) Å3
Z	2	8	2
Reflections collected	53595	68199	63359
Independent reflections	10120 [*R*(int) = 0.0447]	12176 [*R*(int) = 0.0820]	11148 [R(int) = 0.0384]
Completeness (theta)	99.5%	99.5%	99.6%
Goodness-of-fit on F^2	1.054	1.071	1.037
Final *R* indices [*I*>2sigma(*I*)]	*R*1 = 0.0443, *wR*2 = 0.1101	*R*1 = 0.0586, *wR*2 = 0.1541	*R1* = 0.0233, *wR2* = 0.0569
Largest diff. peak and hole	2.960 and -2.914 e.$Å^{-3}$	4.513 and -3.763 e.$Å^{-3}$	1.125 and -1.095 e.$Å^{-3}$

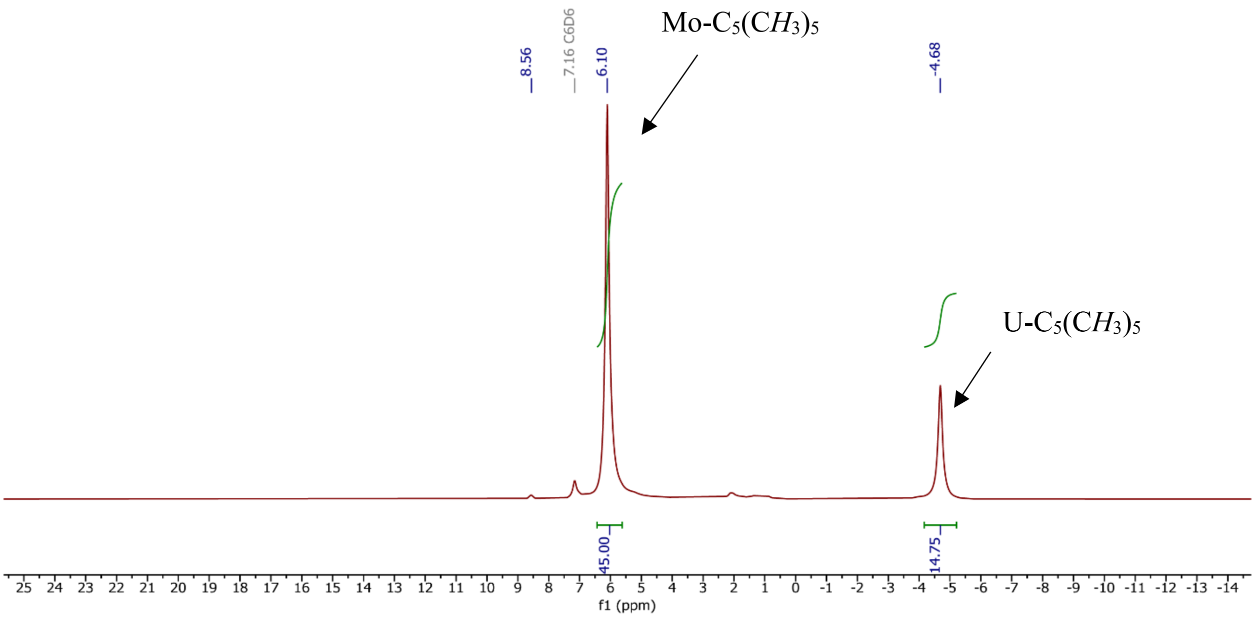

Figure S1. ^{1}H NMR spectrum (400 MHz) of [(Cp*$_3$Mo$_3$S$_4$)Cp*U(OH)] in benzene-*d*$_6$. The trace signal at δ = 8.56 ppm is attributed to methyl protons of Mo-Cp* of free neutral cluster, (Cp*$_3$Mo$_3$S$_4$).

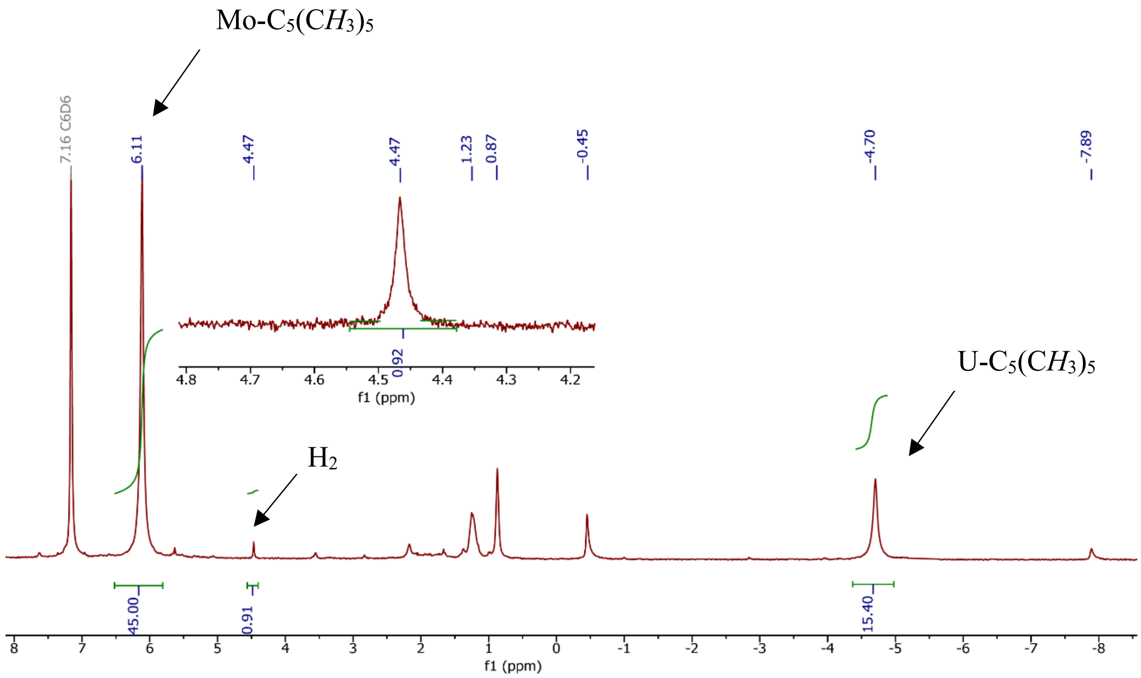

Figure S2. ^{1}H NMR spectrum (400 MHz) of the reaction mixture containing C_6D_6 solution of (Cp*$_3$Mo$_3$S$_4$)Cp*U and benzene-*d*$_6$ solution having slightly more than 1 equivalent of water in THF-d$_8$. Signal located at δ = 4.47 ppm is assigned to H_2 (~80% yield), while signals at δ = -0.45 and -7.89 are attributed to the resonances of methyl protons of Mo-Cp* and U-Cp* of the starting cluster, (Cp*$_3$Mo$_3$S$_4$)Cp*U.

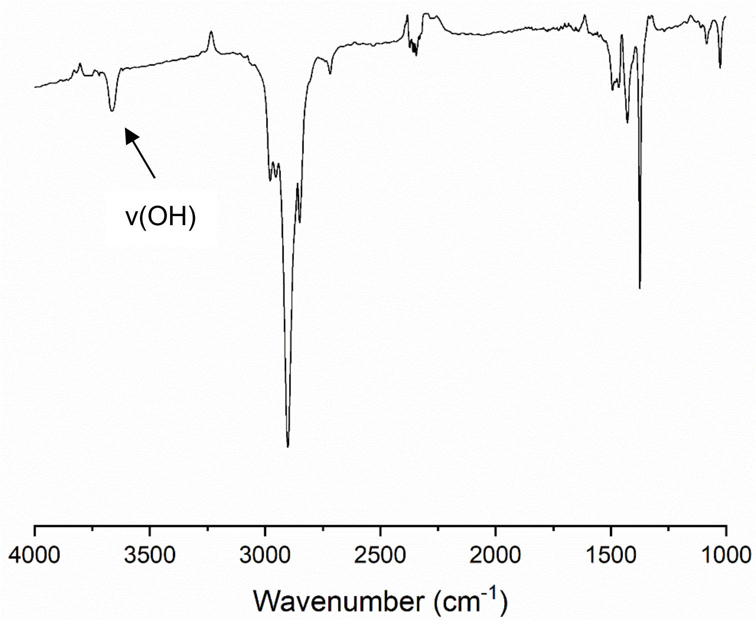

Figure S3. Infrared spectrum of $(Cp^*{}_3Mo_3S_4)Cp^*U(OH)$ collected in benzene-d_6.

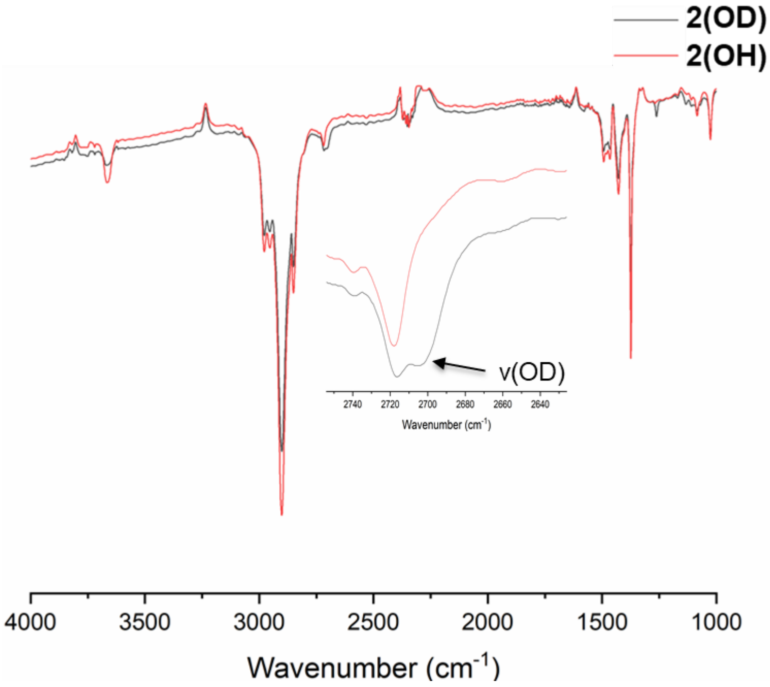

Figure S4. Stacked infrared spectra of $(Cp^*{}_3Mo_3S_4)Cp^*U(OH)$, **2(OH)** and $(Cp^*{}_3Mo_3S_4)Cp^*U(OD)$, **2(OD)** collected in benzene-d_6.

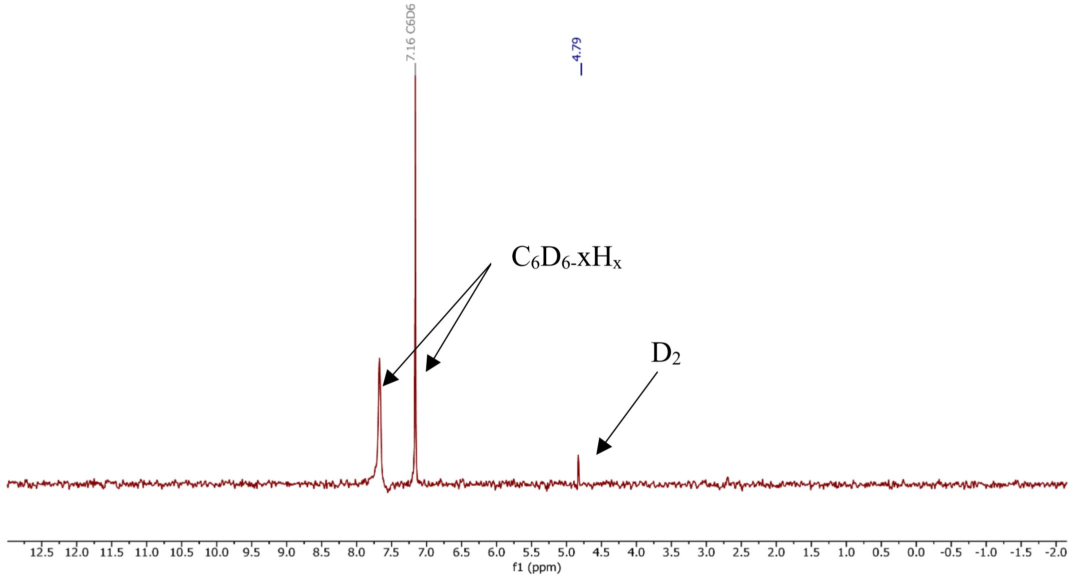

Figure S5. ^{2}H NMR spectrum (400 MHz) of the reaction mixture containing a C_6H_6 solution of ($Cp^*_3Mo_3S_4$)Cp*U and a benzene-d_6 solution with slightly more than 1 equivalent of D_2O in THF. Benzene-d_6 has been added as internal reference; δ = 4.79 ppm is assigned to D_2.

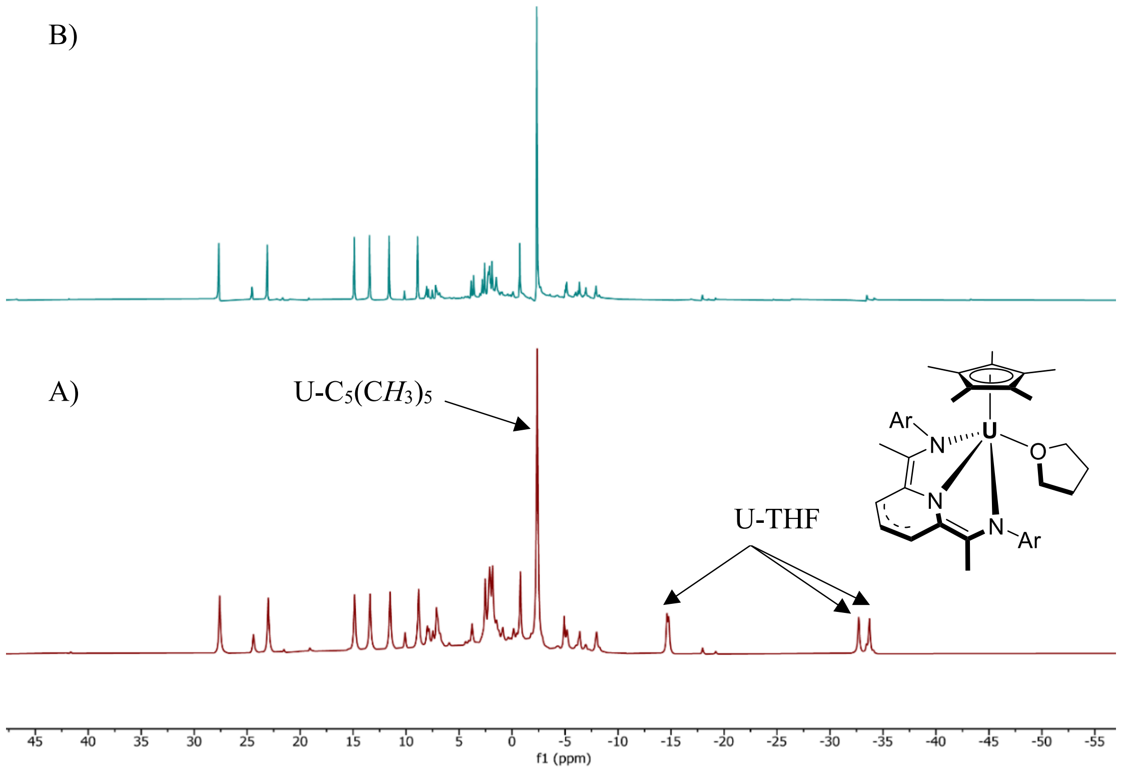

Figure S6. A) ^{1}H NMR spectrum (400 MHz) of (PDI)UCp*(THF) in benzene-d_6; **B**) The ^{1}H NMR spectrum (400 MHz) of the reaction mixture containing benzene-d_6 solution of [(PDI)UCp*(THF)] and benzene-d_6 solution having slightly more than 1 equivalent of water in THF-d_8 (U-THF peaks are assigned as previously reported for this compound).

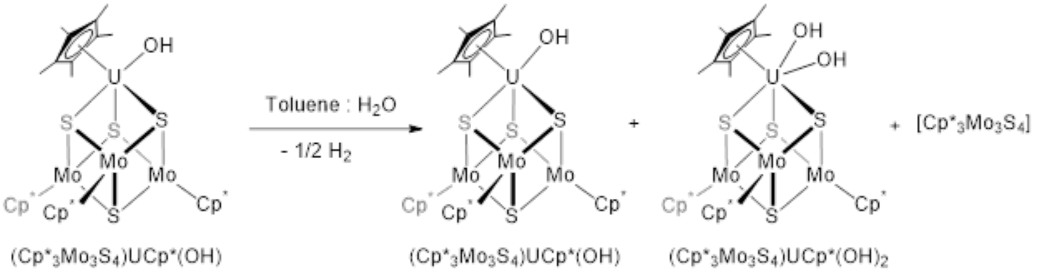

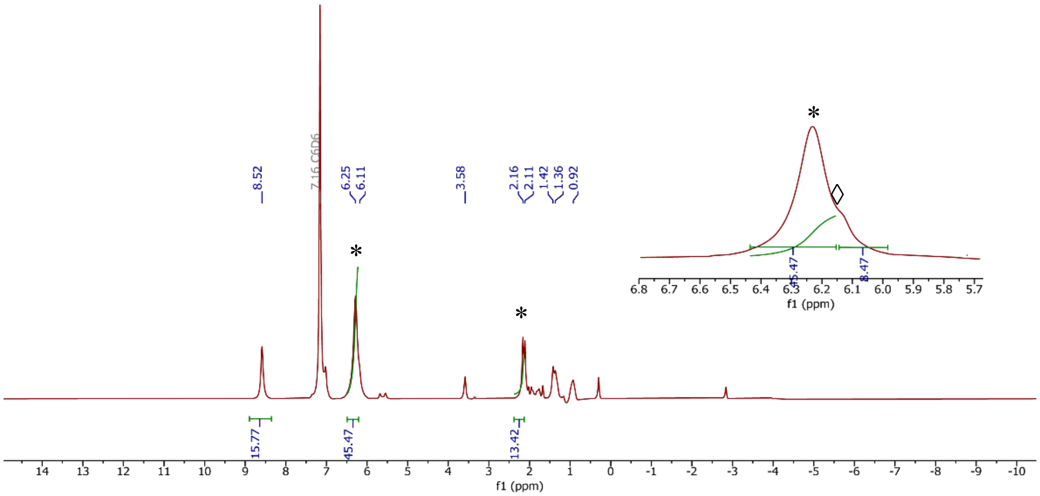

Figure S7. ¹H NMR spectrum (400 MHz) of the crude product obtained from the reaction of $(Cp^*_3Mo_3S_4)UCp^*(OH)$ and toluene-water binary mixture in benzene-d_6 [δ = 8.52 ppm corresponds to the resonance for methyl protons of Mo-Cp* of U-free metalloligand, δ = 6.11 ppm corresponds to resonances for methyl protons of Mo-Cp* signal of $(Cp^*_3Mo_3S_4)UCp^*(OH)$ (◊ (in-set spectrum)), δ = 6.25 ppm and 2.16 ppm assigned to methyl proton resonances of Mo-Cp* and U-Cp* of $(Cp^*_3Mo_3S_4)UCp^*(OH)_2$ (*): Solvent impurities; δ = 3.58 and 1.41 ppm corresponds to THF, δ = 2.11 ppm corresponds to toluene, δ = 0.92 and 1.36 ppm corresponds to pentane]. The ratio of the three products, as determined from the intensity of the methyl protons of Mo-Cp*, is 2:5:1 (U-free $Cp^*_3Mo_3S_4$: bis-hydroxide compound **3** : mono-hydroxide compound **2**). A similar measurement of the crude product obtained from the concentrated solution containing **2** and ~1.5 equivalents of water after storing at -30 °C for a week shows more of the free-cluster with a negligible amount of mono-hydroxide compound **2**. The ratio found was 1:1.3 between compound **3** and uranium-free $Cp^*_3Mo_3S_4$.

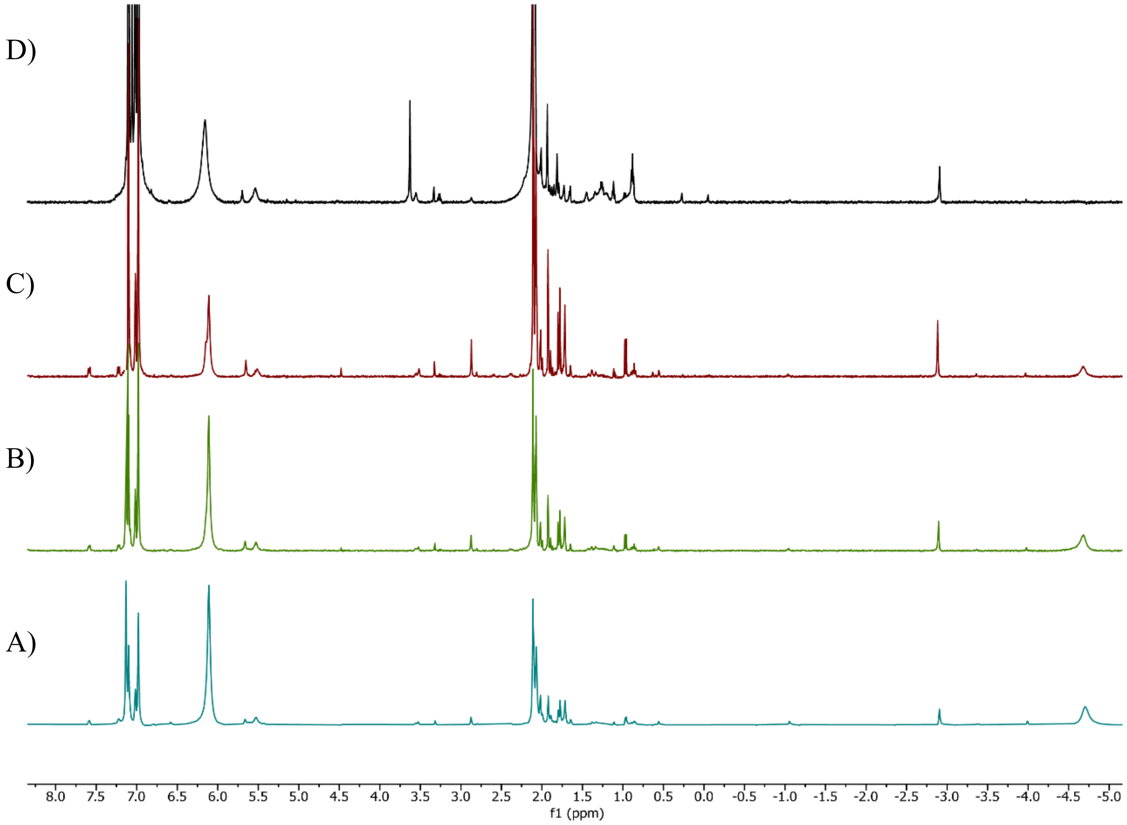

Figure S8. Stacked ^{1}H NMR spectra (400 MHz) of: **A**) ($Cp^*_3Mo_3S_4$)Cp*U(OH), **B**) the reaction mixture containing ($Cp^*_3Mo_3S_4$)Cp*U(OH) and ~0.25 equiv of a water, **C**) the reaction mixture containing ($Cp^*_3Mo_3S_4$)Cp*U(OH) and ~0.5 equiv of a water, and **D**) the crude product obtained from the concentrated solution containing ($Cp^*_3Mo_3S_4$)Cp*U(OH) and ~1.5 equiv of water after storing at -30 °C for a week, in toluene-d_8.

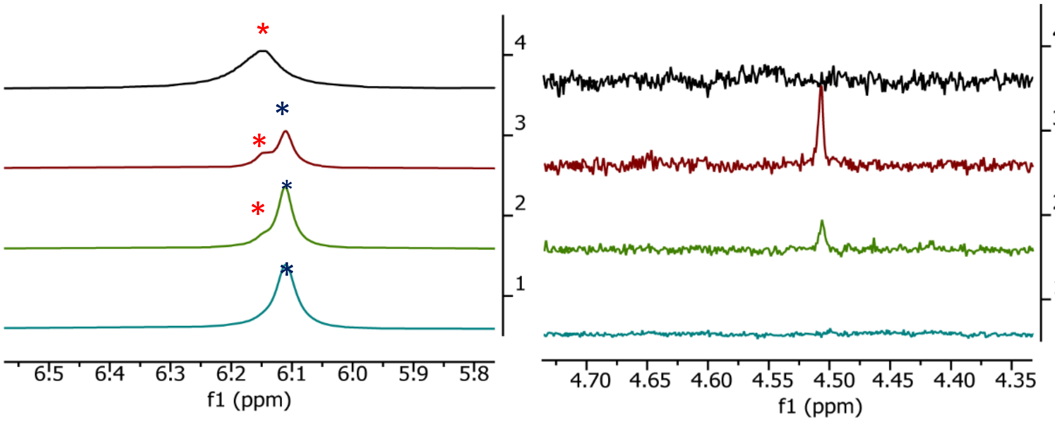

Figure S9. The relevant zoomed-in portion of the spectra provided in **Figure S8**. The * signal on the left-part of the spectra represents the Mo-Cp* methyl protons of **2**, while * signal represents the Mo-Cp* methyl protons of **3**. The signal on the right part of the spectra represents H_2.

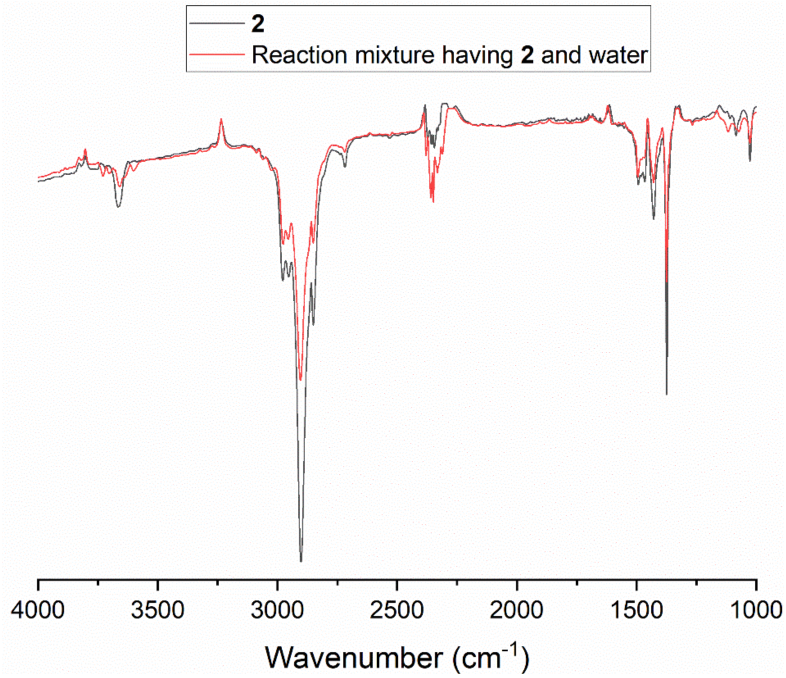

Figure S10. The stacked infrared spectra of ($Cp^*_3Mo_3S_4$)Cp*U(OH), **2**, and the crude reaction mixture containing ($Cp^*_3Mo_3S_4$)Cp*U(OH), **2**, and ~1.5 equivalent of water in toluene-d_8.

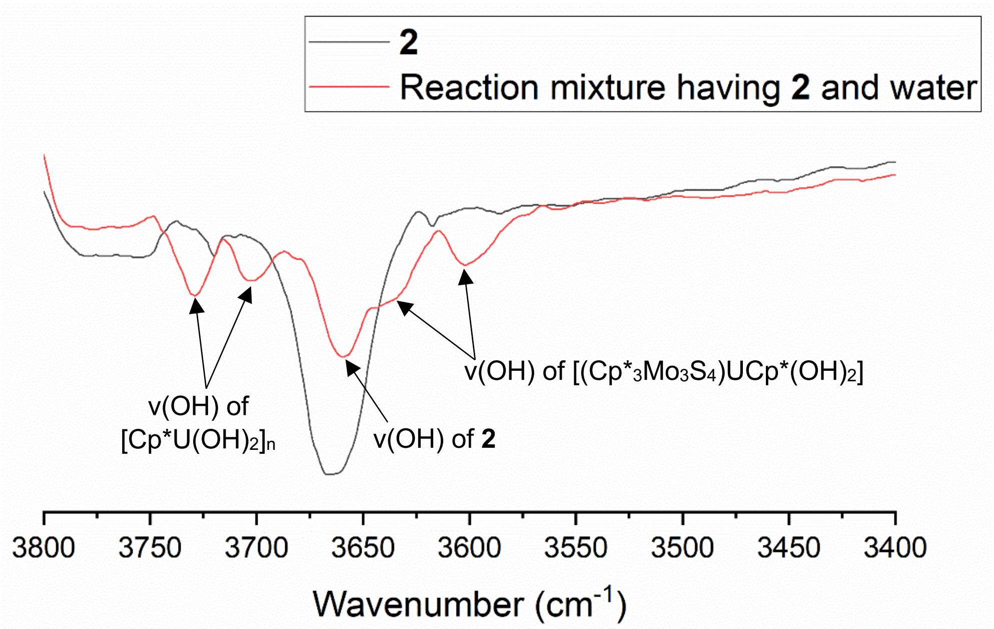

Figure S11. The selected region of *v*(OH) (3400-3800 cm^{-1}) of the stacked spectra shown in **Figure S10** [The *v*(OH) centered at 3668 cm^{-1} corresponds to the mono-hydroxide compound **2,** *v*(OH) centered at 3604 and 3645 cm^{-1} is attributed to the OH groups of bis-hydroxide compound **3**, and *v*(OH) centered at 3704 and 3730 cm^{-1} is attributed to the OH groups of the proposed cluster-free uranium hydroxide compound (Scheme S1)].

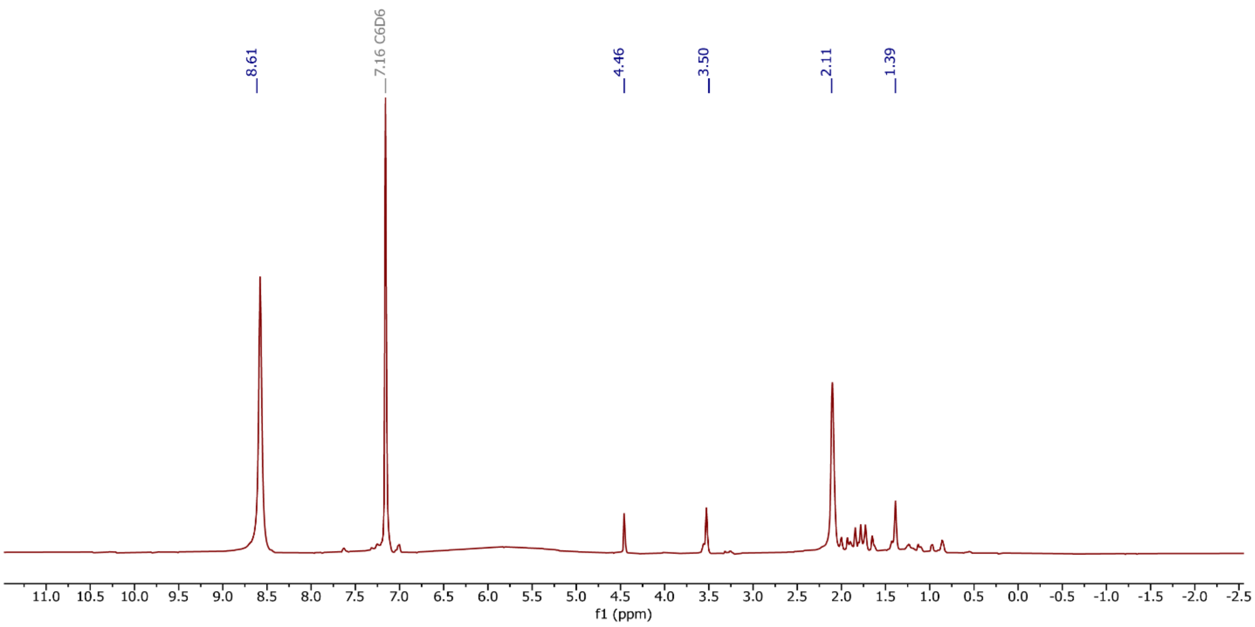

Figure S12. ^{1}H NMR spectrum (400 MHz) of the reaction mixture containing benzene-d_6 solution of $(Cp^*{}_3Mo_3S_4)Cp^*U$ and benzene-d_6 solution having excess of water in THF-d_8. The signal at δ = 8.61 ppm is attributed to the resonance of methyl protons of Mo-Cp* of the free neutral cluster $[(Cp^*{}_3Mo_3S_4)]$, while δ = 4.46 ppm is attributed to H_2; solvent impurities located δ = 3.50 and 1.39 ppm correspond to THF.

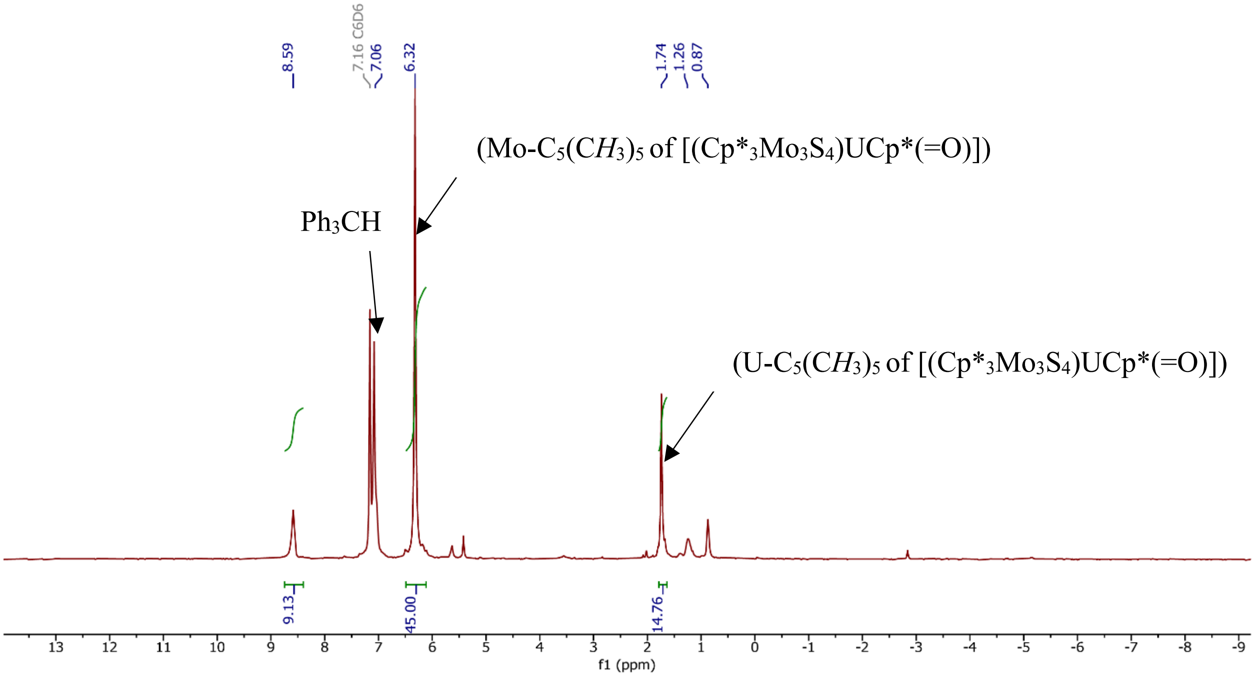

Figure S13. ^{1}H NMR spectrum (400 MHz) of the reaction mixture containing a benzene-d_6 solution of $(Cp^*{}_3Mo_3S_4)Cp^*U(OH)$ and a benzene-d_6 solution with 0.5 equivalent of Gomberg's dimer. The signal at δ = 8.59 ppm is attributed to the resonance of methyl protons of Mo-Cp* of the free neutral cluster $(Cp^*{}_3Mo_3S_4)$, while δ = 7.06 ppm is attributed to the by-product of Gomberg's dimer after abstracting the hydrogen atom (Ph_3CH).

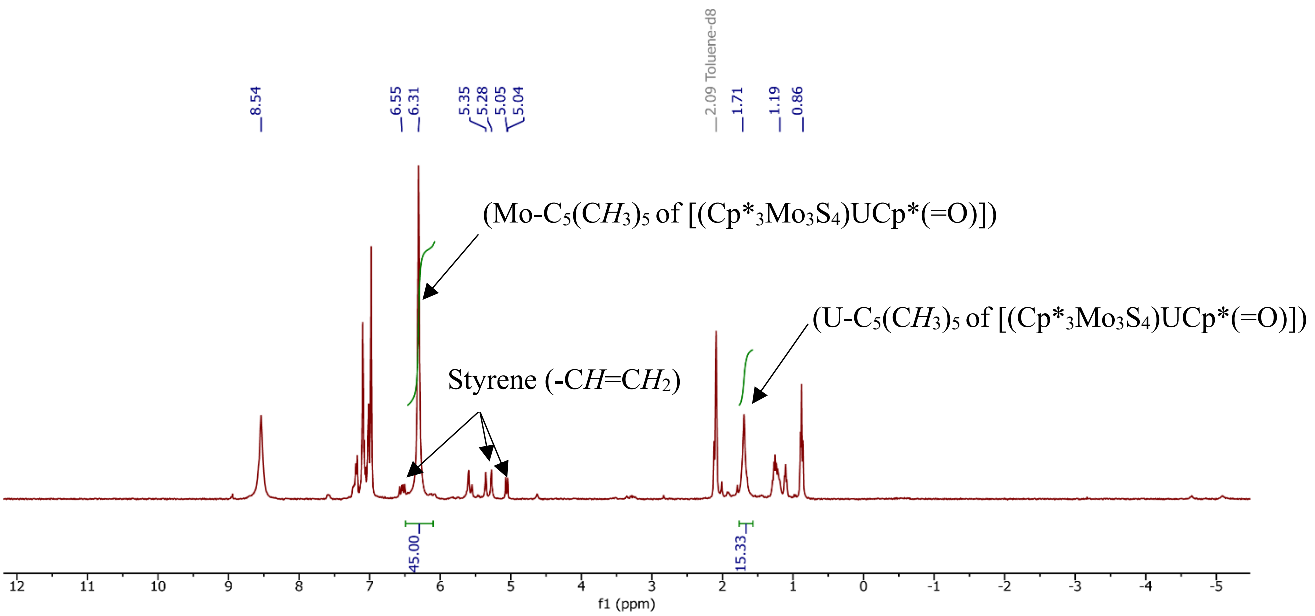

Figure S14. ^{1}H NMR spectrum (400 MHz) (-10 °C) (with slow relaxation time) of the reaction mixture containing a toluene-d_8 solution of $(Cp^*_3Mo_3S_4)Cp^*U$ and a toluene-d_8 solution with 1 equivalent of styrene oxide. The signal at $\delta = 8.54$ ppm is attributed to the resonance of methyl protons of Mo-Cp* of free neutral cluster, $(Cp^*_3Mo_3S_4)$.

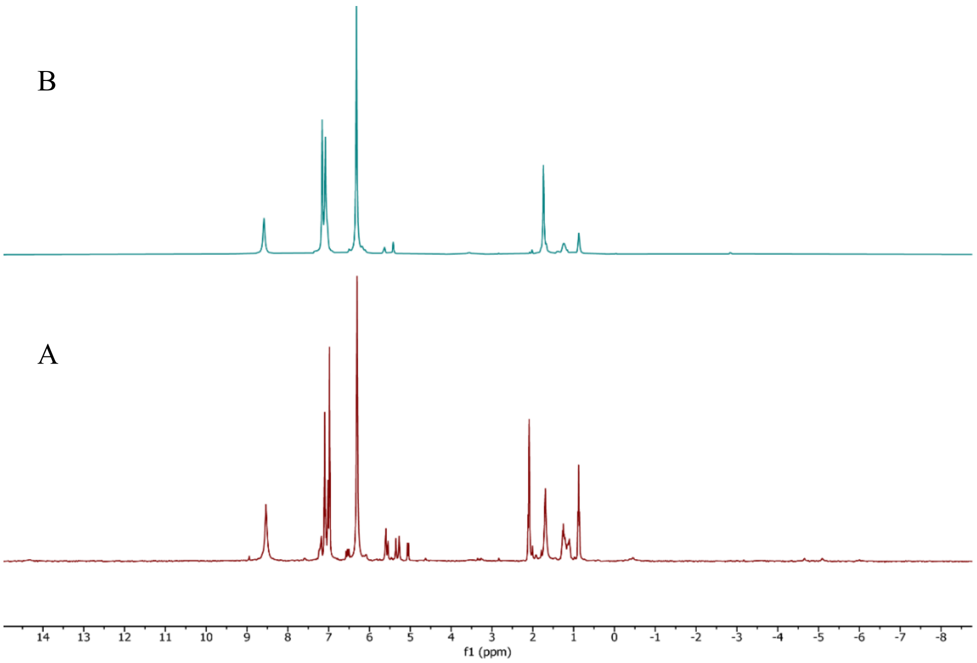

Figure S15. Stacked ^{1}H NMR spectra (400 MHz) of **A**) the reaction mixture containing a Toluene-d_8 solution of $(Cp^*_3Mo_3S_4)Cp^*U$ and a toluene-d_8 solution with 1 equivalent of styrene oxide; **B)** the reaction mixture containing a C_6D_6 solution of $(Cp^*_3Mo_3S_4)UCp^*(OH)$ and a benzene-d_6 solution with 0.5 equivalent of Gomberg's dimer.

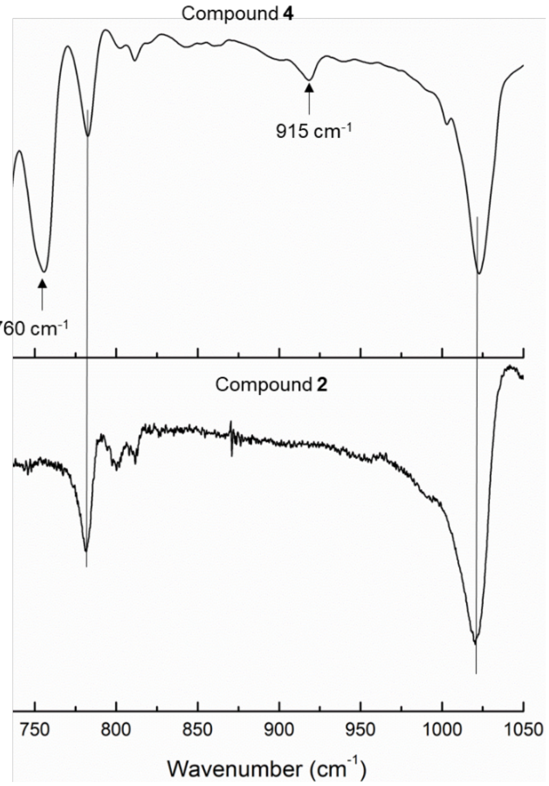

Figure S16. Stacked infrared spectra of $(Cp*_3Mo_3S_4)UCp*(OH)$ (**2**) and $(Cp*_3Mo_3S_4)UCp*(=O)$ (**4**) collected via ATR. Although the mono-hydroxide species **2** is stable enough to perform all the required characterizations, the uranium oxo species **4** is highly unstable. We observed rapid decomposition in ^{1}H NMR while performing the synthesis via either pathway, even at low temperatures (hydrogen atom transfer using Gomberg's dimer or oxygen atom transfer using styrene oxide), which precludes further characterization of the oxo compound **4**. In this figure, we have attempted to characterize the uranium oxo compound **4** using FT-IR spectroscopy and observed bands at 760 cm^{-1} in both products obtained from the HAT and OAT reactions, along with a weak band at 915 cm^{-1} in the product obtained from the HAT reaction. These bands were absent in compound **2**. Comparing these with uranium oxo compounds reported in the literature suggests a close resemblance to UO_2 (775 cm^{-1}) and UO_2^+ (952 cm^{-1}). This observation is consistent with decomposition observed by ^{1}H NMR spectroscopy. We argue that the limitations of our model in stabilizing uranium oxo or uranyl derivatives, possibly stemming from weaker $U\text{-}S_{surf}$ bonds compared to other reported analogous compounds featuring stronger U-X (X = N, O) bonds.

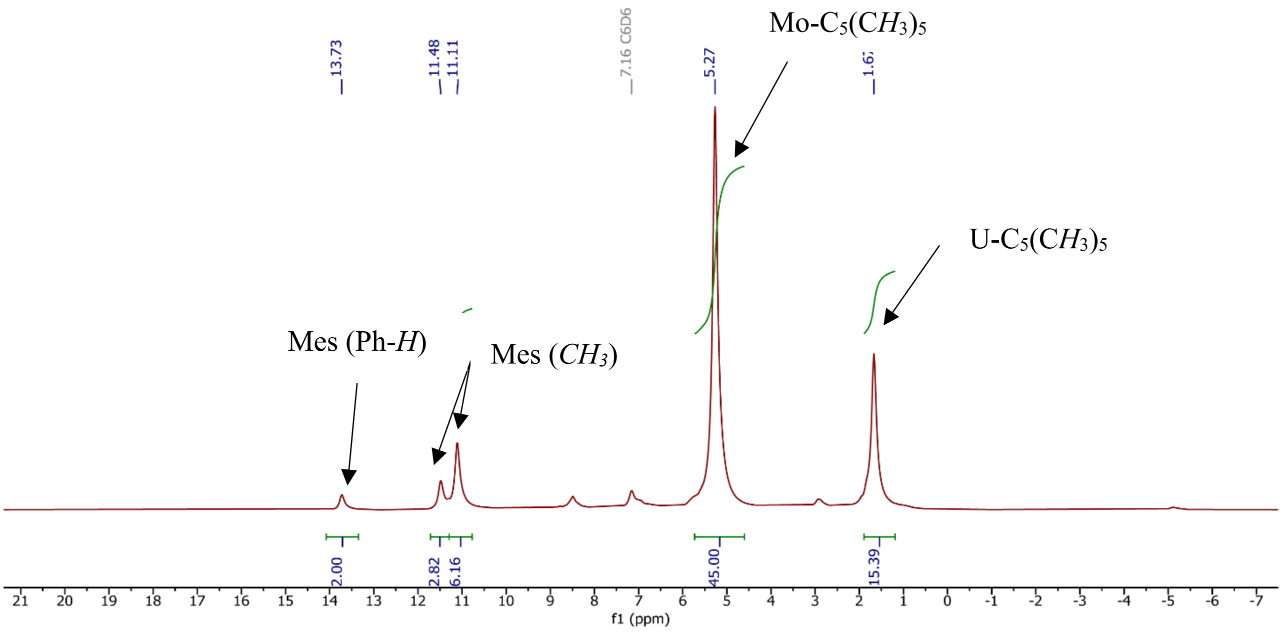

Figure S17. ^{1}H NMR spectrum (400 MHz) of $(Cp^*_3Mo_3S_4)UCp^*(=NMes)$ in benzene-d_6.

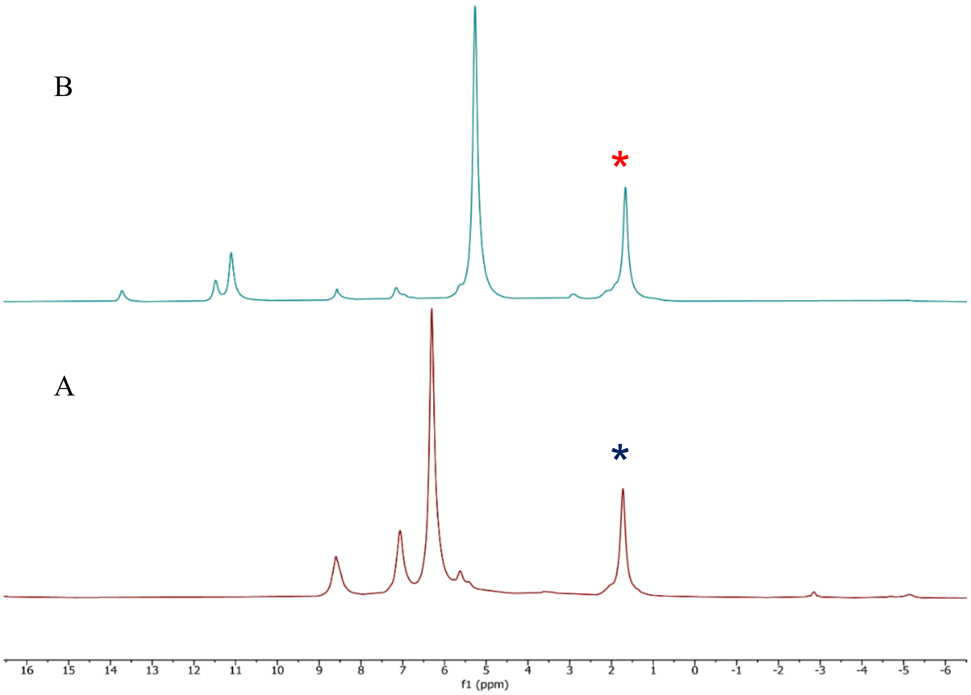

Figure S18. Stacked ^{1}H NMR spectra (400 MHz) of **A**) the reaction mixture containing a C_6D_6 solution of $(Cp^*_3Mo_3S_4)Cp^*U(OH)$ and a benzene-d_6 solution with 0.5 equivalent of Gomberg's dimer **B)** $(Cp^*_3Mo_3S_4)UCp^*(=NMes)$ in benzene-d_6; * is assigned to the resonance of U-Cp* methyl protons of $(Cp^*_3Mo_3S_4)Cp^*U(=O)$ and * is assigned to the resonance of U-Cp* methyl protons of $[(Cp^*_3Mo_3S_4)Cp^*U(=NMes)$.

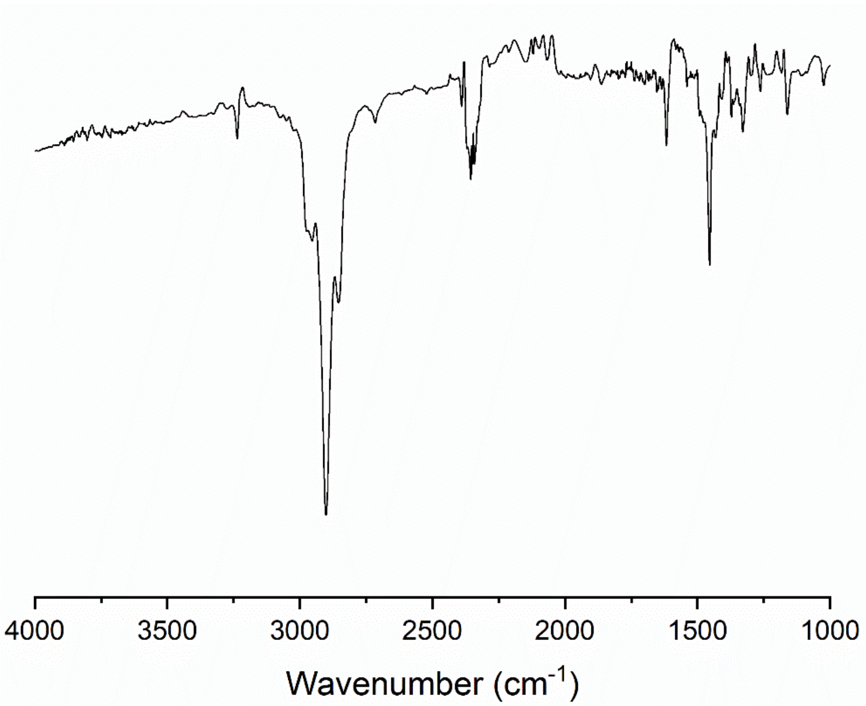

Figure S19. The infrared spectrum of $(Cp^*{}_3Mo_3S_4)Cp^*U$ in benzene-d_6.

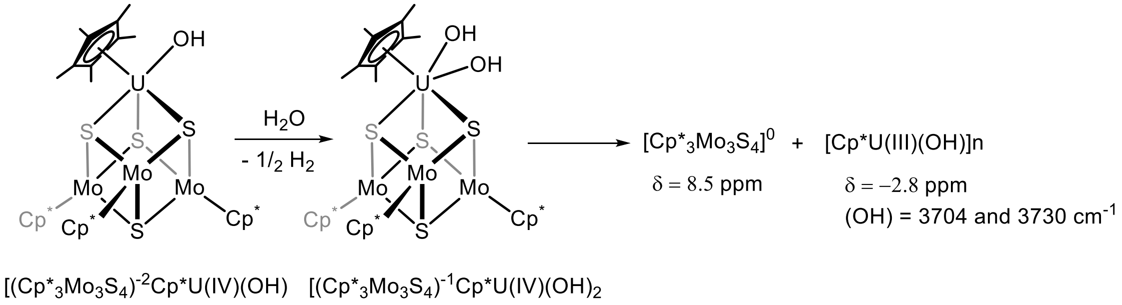

Scheme S1. A possible decomposition pathway for the bis-hydroxide cluster, $(Cp^*{}_3Mo_3S_4)Cp^*U(OH)_2$, **3,** as evidenced from ^{1}H NMR and IR spectroscopy.
